# Theory of radiologist interaction with instant messaging decision support tools: A sequential-explanatory study

**DOI:** 10.1371/journal.pdig.0000297

**Published:** 2024-02-26

**Authors:** John Lee Burns, Judy Wawira Gichoya, Marc D. Kohli, Josette Jones, Saptarshi Purkayastha

**Affiliations:** 1 Department of Radiology & Imaging Sciences, Indiana University School of Medicine, Indianapolis, Indiana, United States of America; 2 Department of BioHealth Informatics, Indiana University Luddy School of Informatics, Computing, and Engineering, Indianapolis, Indiana, United States of America; 3 Department of Radiology and Imaging Sciences, Emory University School of Medicine, Atlanta, Georgia, United States of America; 4 Department of Radiology and Biomedical Imaging, University of California San Francisco, San Francisco, California, United States of America; Drexel University, UNITED STATES

## Abstract

Radiology specific clinical decision support systems (CDSS) and artificial intelligence are poorly integrated into the radiologist workflow. Current research and development efforts of radiology CDSS focus on 4 main interventions, based around exam centric time points–after image acquisition, intra-report support, post-report analysis, and radiology workflow adjacent. We review the literature surrounding CDSS tools in these time points, requirements for CDSS workflow augmentation, and technologies that support clinician to computer workflow augmentation. We develop a theory of radiologist-decision tool interaction using a sequential explanatory study design. The study consists of 2 phases, the first a quantitative survey and the second a qualitative interview study. The phase 1 survey identifies differences between average users and radiologist users in software interventions using the User Acceptance of Information Technology: Toward a Unified View (UTAUT) framework. Phase 2 semi-structured interviews provide narratives on why these differences are found. To build this theory, we propose a novel solution called Radibot—a conversational agent capable of engaging clinicians with CDSS as an assistant using existing instant messaging systems supporting hospital communications. This work contributes an understanding of how radiologist-users differ from the average user and can be utilized by software developers to increase satisfaction of CDSS tools within radiology.

## Author summary

There is a need for human-machine interfaces between radiologists and clinical decision support systems (CDSS). Within the variety of systems radiologists interact with, there is no best fit for CDSS presented in the literature. After reviewing current literature surrounding CDSS use in healthcare and radiology, we propose a novel solution—a conversational agent capable of engaging clinicians as a team member using existing instant messaging systems supporting hospital communications.

We test the acceptance of this intervention using the User Acceptance of Information Technology: Toward a Unified View (UTAUT) framework in survey and interview formats. Within our sample group, we found that radiologists have a high intent to use and a positive attitude towards this intervention. Our sample of radiologists deviated from the standard user UTAUT expects, suggesting that radiologist’s acceptance of software tools differs from the standard user. This work builds a theory of radiologist-decision support tool interaction that may be useful for software developers and systems integrators.

## 1 Introduction

Clinical decision support systems (CDSS) are software designed to enhance clinical decision making, capable of combining clinical knowledge bases and data to provide suggestions for patient care [1]. Radiology domain specific CDSS applications are poorly integrated into the radiologist workflow [2]. In 2017, Dreyer and Geis described a transition in radiology moving towards integrating Artificial Intelligence (AI) into the radiologist workflow. "In the past, radiology was reinvented as a fully digital domain when new tools, PACS and digital modalities, were combined with new workflows and environments that took advantage of the tools. Similarly, a new cognitive radiology domain will appear when AI tools combine with new human-plus-computer workflows and environments." They describe the concept of a "Centaur Radiologist" as a physician utilizing AI-augmented CDSS workflows to increase efficiency [3]. We expand this term as “future radiologist,” inclusive of non-AI techniques in CDSS.

However, the future radiologist concept will not happen if the tools are poorly integrated, with cumbersome human-computer interfaces [4]. Deliberate and sustained effort by using inter-disciplinary knowledge from human-centered computing, psychology, cognitive sciences, and medicine is required to build CDSS for the future radiologist [5]. In this work we create a basis of knowledge in the theory of radiologist-decision tool interaction using a sequential explanatory study design. The study consists of 2 phases, the first a quantitative survey and the second a qualitative interview study. The phase 1 survey identifies differences between average users and radiologist users in software interventions using the User Acceptance of Information Technology: Toward a Unified View (UTAUT) framework [6]. Phase 2 semi-structured interviews provide narratives on why these differences are found. To build this theory, we propose a novel solution called *Radibot*—a conversational agent (CA) capable of engaging clinicians with CDSS as an assistant using existing instant messaging (IM) systems supporting hospital communications. This work contributes an understanding of how radiologist-users differ from the average user and can be utilized by software developers to increase satisfaction of CDSS tools within radiology.

### 1.1 Background

We expect that the future radiologist will routinely interact with CDSS at each stage of their workflow. section C.1 in S1 Appendix includes an extended background of radiology CDSS including standards and features of radiology workflow and associated systems, and an overview of the backend workflow engines that support radiology CDSS tools. We designed Radibot for diagnostic radiologists, with interventions at each of the following time-points: after image acquisition, during report creation, after report creation, and between studies. A brief overview of existing interventions in each time point follows. Section C.1 in S1 Appendix includes an extended background of interventions at these time-points.

- After Image Acquisition—radiologists combine a variety of data to make interpretations of images. Interventions include Computer-Aided Detection (CAD), where regions of interest are highlighted for later interpretation; Computer-Aided Diagnosis (CADx), where the computer presents a diagnosis but does not necessarily highlight regions of interest; and patient history/metadata presentation. These interventions generally function within the tool radiologists use to view images, the Picture Archiving and Communication System (PACS), though some will interface with the Radiology Information System (RIS) that houses scheduling/billing/patient metadata, Voice Recognition system (VR) used for report dictation, or in an external purpose built clients [7–17].
- During Report Creation–these interventions surround embedding evidence-based guideline processes during dictation and are found within VR. Guidelines are navigated using drill-through commands or natural language processing (NLP) of the dictation to generate report text [18,19].
- After Report Creation—In most RIS, reports are stored as unstructured text. Interventions in post-report analysis include extracting categorical data, automating radiologist-clinician communication, and quality improvement systems. By generating summative report metadata, these interventions enable context-switching and reduce fatigue when a radiologist is asked to return to a finished report [20–32].
- Between Studies–existing adjacent to radiologist workflow, these interventions influence decision making at an individual or business level and consist of workflow-prioritization, management, and feedback tools. These tools utilize metadata found in Health Level 7 (HL7) or Digital Imaging and Communications in Medicine (DICOM) messages. Users interface with them outside of clinical systems, IE. web dashboards, or they are integrated into PACS/RIS/VR presentation layers [33–40].

Diagnostic radiologist’s clinical work is mostly completed using systems, including PACS, RIS, and VR, with nearly every interaction being digitally augmented [41]. Given the mostly digital clinical workflows, radiology specific CDSS implementations are uniquely positioned to provide support and affect change. Radiology specific guidelines for "advisor systems" were laid out by Teather et al. in 1985, while Khorasani in 2006 provides features for the development of clinical decision support systems [42,43]. Outside of radiology, CDSS are built following the Ten Commandments for Effective Clinical Decision Support: Making the Practice of Evidence-Based Medicine a Reality. These 10 commandments summarize elements authors found critical for successful implementation of decision support in clinical workflows–

1. “Speed is everything
2. Anticipate needs and deliver in real time
3. Fit into the users workflow
4. Little things can make a big difference (usability of CDSS tools)
5. Recognize that physicians will strongly resist stopping
6. Changing direction is easier than stopping
7. Simple interventions work best
8. Ask for additional information only when you really need it
9. Monitor impact, get feedback, and respond
10. Manage and maintain your knowledge-based systems” [44].

Commandments 2, 3, 7, 10, and 1 –anticipate needs, fit into user workflow, simple interventions, knowledge system maintenance, and speed–appear with a higher frequency when aligned with radiology specific guidance. An alignment of the general CDSS and radiology specific CDSS guidelines are found in Table 1. Differences in CDSS priorities underscore the need for more research in this area and are mapped to UTAUT concepts and the hypotheses for phase 1.

**Table 1:** Commandments compared to published considerations.

Commandments^a^/Considerations	Speed is Everything	Anticipate Needs and Deliver in Real Time	Fit Into the Users Workflow	Little Things Can Make a Big Difference	Recognize that Physicians Will Strongly Resist Stopping	Changing Direction is Easier than Stopping	Simple Interventions Work Best	Ask for Additional Information Only When You Really Need It	Monitor Impact, Get Feedback, and Respond	Manage and Maintain Your Knowledge-Based Systems
"The system must follow the usual working practices of the clinician and must not appear to usurp his/her position." ^b^			X		X					
"The system must have a good, adaptable user interface which uses clinical terminology, and is able to give help on demand." ^b^	X	X	X				X			
"Diagnostic advice (if sought) must be ’calibrated’ so that uncertainties output by the system may be interpreted in terms of the incidence of errors. Explanation/justification of conclusions should be available on demand." ^b^		X						X		X
"For the system to be used, rather than just usable, it must offer more than just simple diagnostic advice, and these other facilities should be available independently of diagnostic advice." ^b^		X	X							X
Time sensitivity, with a preference for real time CDSS. ^c^	X	X								
Brevity in providing salient information to answer the clinical question asked and link to evidence. ^c^			X	X			X			
Recommend action and provide descriptive reasoning and actionable interventions ^c^						X	X			
CDSS must use up to date evidence-based medicine ^c^										X
CDSS should improve productivity, efficiency, quality, and safety. ^c^									X	

^*a*^ 10 commandments from Bates et al. [44].

^*b*^ Considerations quoted from Teather et al. [42]

^*c*^ Considerations paraphrased from Khorasani [43]

Other frameworks exist for testing usability and user experience for software design in survey form. However, UTAUT is unique in the number of constructs it can capture quickly. UTAUT was developed as a theoretical model that combines measures like the System Usability Scale or Technology Acceptance Model. Other measures can capture intent to use, but do not create the linkages to potential moderating factors of interest for this study including expected effort, expected performance, anxiety, age, and experience with similar tools [6]. UTAUT is an accepted and comprehensive model for technology adoption [6,45–47]. Within the UTAUT framework we focused on the following factors below, as appropriate the UTAUT concepts are linked to the CDSS commandments described above [5]:

- Behavioral intention to use the system
  ○ Positive behavioral intent indicates stronger intent to use the system if created
- Attitude toward using the technology
  ○ Positive attitude indicates positive reaction to using the application
  ○ Performance Expectancy
  ○ Positive expected performance indicates perceived performance gains
  ○ 1. Speed is everything
  ○ 2. Anticipate Needs and Deliver in Real Time
  ○ 5. Recognize that physicians will strongly resist stopping
  ○ 7. Simple interventions work best
- Effort Expectancy
  ○ Positive expected effort indicates perceived increased ease of use of the system compared to similar applications
  ○ 3. Fit into the user’s workflow
  ○ 4. Little things can make a big difference
  ○ 6. Changing Direction is Easier than Stopping
  ○ 8. Ask for Additional Information Only When You Really Need It
- Anxiety
  ○ Positive anxiety indicates increasing negative emotions towards the system
  ○ 5. Recognize that physicians will strongly resist stopping

### 1.2 Instant Messaging and Conversational Agents (CA) in Healthcare

IM is found throughout the healthcare enterprise, including in disease management, patient-clinician interactions, medical education, among patient populations and workforce members for extra-clinical activities. IM can be inclusive of voice, video calling, and file sharing [48]. Extra-clinically, IM tools facilitate socialization, catharsis, and professional connectiveness functionalities when applied in clinical settings [49,50]. IM is asynchronous and short-form, leading to advantages over other communication methods, particularly in the area of articulation work—answering medical questions, coordinating logistics, addressing social information for patients, and querying staff/equipment locations or status [51]. IM is integrated into many PACS, RIS, and VR, serving many purposes within radiology including care discussions and facilitating remote tele-radiology communications [30,52–62].

CA are natural language human-machine interfaces capable of synthesizing a variety of information and conversing in less programmatic/fixed ways than other language interfaces like chatbots. CA can apply 4 methods for negotiating user interactions: immediate, negotiated, mediated, and scheduled [63]. Consumer health care CA are currently scheduling appointments, providing basic symptom identification and recommendation, and assisting with long term care such as sensor monitoring/alerting and medication reminders [64]. Most healthcare CA are built for patients (interview, data collection, or telemonitoring), while clinician focused CA are designed around data collection [65]. Other efforts in clinician focused CA include interpreting spoken language into clinical facts and drug interaction/alternative drug recommendation systems [66–68]. IM impact on task completion is not fully understood, especially in the context of automated IM interventions. There is evidence that non-relevant messages can increase or reduce task completion times depending on the message initiator; at a cost of quality of the task output [69]. Disruptiveness of IM specific interventions is reduced when IM are relevant to the task being completed or if delivered at time-points that fit the user’s workflow [70]. IM interactions among a professional workforce are found to support task completion, accuracy, and quality of outcomes [69]. Historically CA were powered by rules-based systems or ‘small’ AI language models, while modern CA like ChatGPT are using Large Language Models (LLM) [64–68,71–74].

## 2 Methods

### 2.1 Population

Our study population consists of radiologists– 112 attendings and 62 resident or fellow trainees at a large academic health system. Our population is acquired through convenience sampling. Of 174 possible participants, 98 responded affirmative that they would complete the survey and 3 that they did not want to participate. 39 participants responded that they would complete an interview and 11 responded that they would participate in the survey but not the interview. In total, 88 surveys were submitted, and 23 interviews were conducted.

### 2.2 Survey

An electronic survey was created using Qualtrics [75] that collects population composition and quantitative data surrounding intervention feasibility, usability, and acceptance. We chose to not utilize questions in social influence, facilitating conditions, and self-efficacy due to applicability to a prospective study of a tool not yet implemented in practice. A full listing of UTAUT questions by construct and factor are found on EDUTECH’s Wiki [47]. Due to respondent time constraints we chose to utilize 12 of 19 questions in the chosen constructs, with each construct having at least 2 questions asked [47]. Questions were eliminated if they were not relevant to a system that does not yet exist (Example: Working with the system is fun). Construct validity and reliability is confirmed with structured equation modeling (SEM).

**Fig 1:**
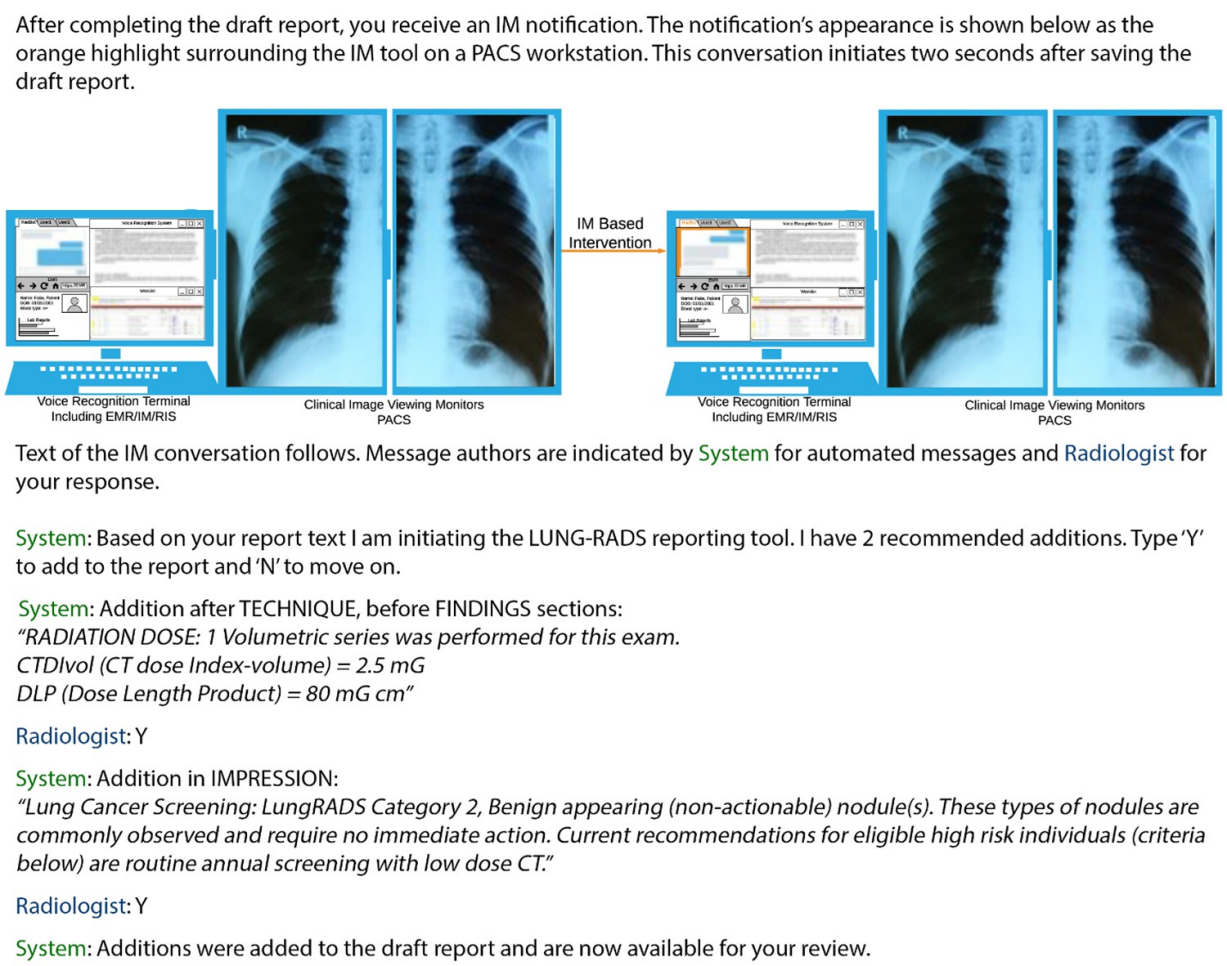
Sample PACS workstation before/after IM based intervention, and details of intervention presented to survey takers. **Source images for Lung X-Ray [76], Report [77], and IM transaction [78]. LUNG-RAD scenario and output text [79]**.

The survey in full is included in Section A.1 in S1 Appendix. Fig 1 highlights the intervention and proposed capabilities.

### 2.3 Interview

**Fig 2:**
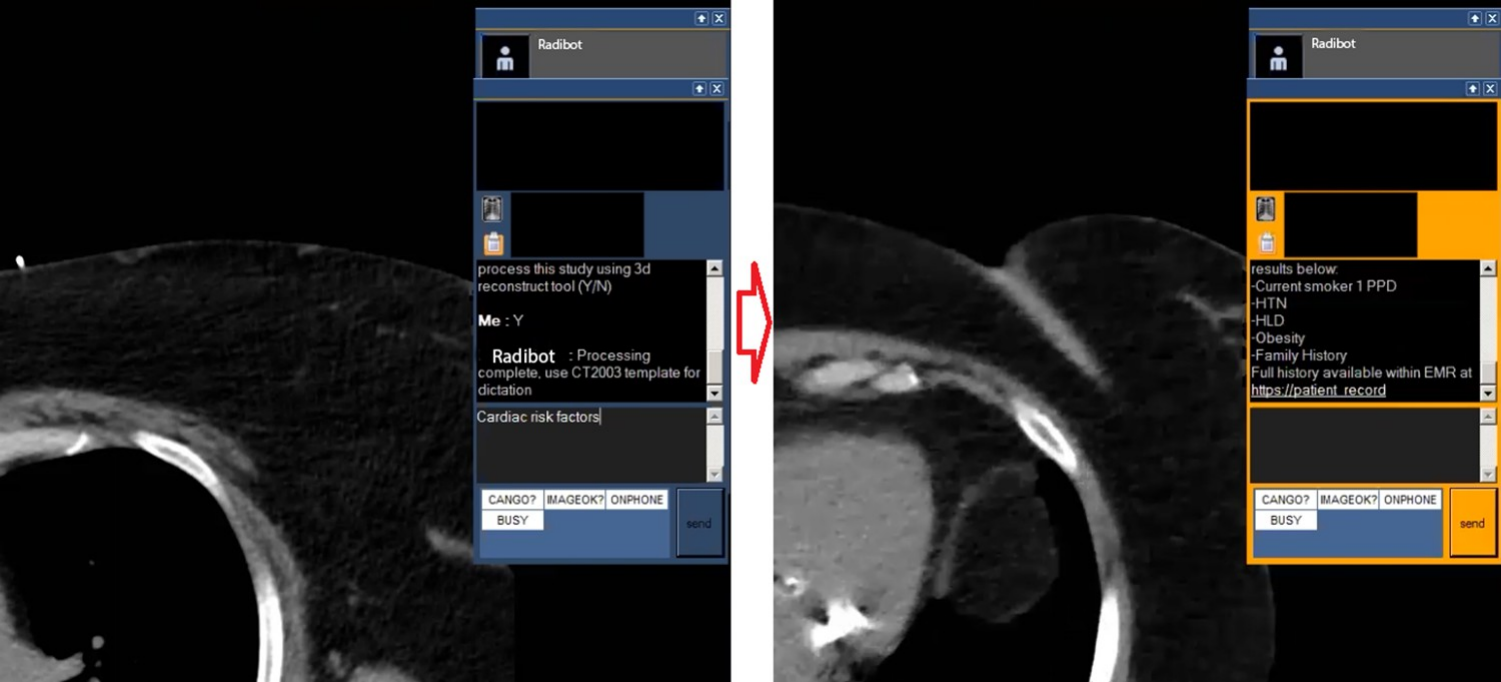
Capture from Video 2 highlighting radiologist query and Radibot response.

Using the research statements developed with the survey (Table A.5 in S1 Appendix), we generated hypotheses and began developing the semi-structured interviews. As we did not have a working system, we prototyped 5 interventions and created video examples of each to use during the interview. Fig 2 highlights what these videos looked like during a demo. The videos highlighted interventions during each workflow time point in the following ways:

- After Image Acquisition
  ○ Video 1 –Radibot identifies potential for 3d reconstruction, asks radiologist permission to process, and then suggests the correct VR template.
- During Report Creation
  ○ Video 2 –Radiologist engages Radibot to query the Electronic Medical Record (EMR) for cardiac risk factors. Radibot performs this query as the radiologist returns to reviewing images, then returns all risk factors that meet these criteria.
  ○ Video 3 –Radibot identifies VR dictation of left adrenal nodule then engages radiologist in stepping through adrenal nodule flow chart. Completion of the flowchart inserts guideline recommend text and citation into report.
- After Report Creation
  ○ Video 4 –Based on text of report, Radibot engages radiologist for follow up communication.
- Between Studies
  ○ Video 5 –Radibot presents possible studies for radiologists to engage with, removing the need to navigate the worklist. Includes suggestions of cross coverage of busier worklists and high priority studies.

An interview guide was created (Figure B.1 in S1 Appendix) following the UTAUT framework. The guide begins with video 1, loops through each video asking the same questions, then has a set of questions after all videos have completed. A portable interview setup was created consisting of one laptop, a 4k portrait monitor mimicking a diagnostic monitor, and a microphone for collecting audio. Interviews occurred in offices/conference rooms located near interview candidates normal work locations. Subjects were presented with consent and informed that no names would be utilized during the interview for confidentiality. Zoom was utilized to record the screen and interview narrative to the laptop [80].

39 survey participants responded that they would complete an interview. 23 interviews occurred before the research team agreed that response saturation was achieved. Interviews were transcribed using Otter.AI, then a research assistant and study team member reviewed each video separately and corrected any transcription errors [81]. Transcriptions were downloaded in docx format, then loaded into ATLAS.ti 9.0.19.0 for qualitative analysis. The study team created labels for text analysis (Table B.2 in S1 Appendix) and linked these by semantic domain (UTAUT construct). 2 research assistants were hired and trained by the study team to annotate interview text using ATLAS.ti. The research assistants separately annotated interview 1, then the study team reviewed and provided additional guidance. They then separately annotated the remaining interview narratives, and the annotated narratives were merged, and inter-rater agreement is measured. Because semantic domains are established and we did not segment quotes in advance, Krippendorff’s CU Alpha is utilized to measure semantic domain agreement by quote. An overall agreement level of α ≥ .8 is set for all documents [82]. A second round of interviews was planned if text analysis was finding new semantic linkages–defined as quotes with more than 1 label linking constructs together. Any individual interview presented no new semantic linkages differing from the remaining interviews, confirming saturation was reached.

## 3 Results

### 3.1 Survey data analysis

Resulting data was downloaded from Qualtrics in Comma Separated Values (CSV) format and analyzed using Excel. Irrelevant metadata fields were removed. A total of 88 survey responses were used for analysis, representing 50.6 percent of the total sample population. After removing 4 outliers that took over an hour to complete the survey, average completion time was found to be 6 minutes and 45 seconds. Raw survey data is available in S2 Survey Data.

Qualitative questions were bucketed into numbers ranging from 0–5 (IE 0 to 5 years = 1; 5 to 10 years = 2; etc.). A full set of questions, response bucketing, and UTAUT constructs are included the Table A.2 in S1 Appendix. Summary data surrounding survey responses used in the analysis are listed in Table 2.

**Table 2:** Summary data for Likert scale questions in survey responses, generated using SmartPLS v. 3.2.9.

Question	Mean	Median	Min	Max	Standard Deviation	Excess Kurtosis	Skewness
**Years Practiced–Q1**	2.33	2	1	5	1.43	-0.93	0.66
**Years Old–Q2**	2.52	2	1	5	0.94	-0.51	0.32
**Radiologist Tools–Q4**	2.33	2	1	5	0.98	-0.16	0.43
**Consumer IM–Q5**	2.49	2	1	5	0.77	0.56	1.19
**Clinical IM–Q6**	3.02	3	1	5	1	-0.61	0.38
**Conversational Agents–Q7**	3.13	3	1	5	1.08	-0.63	0.48
**Performance Expectancy 1 –Q11**	3.73	4	2	5	0.81	-0.35	-0.26
**Effort Expectancy 1 –Q12**	3.92	4	2	5	0.55	1.57	-0.45
**Attitude Toward Using Technology 1 –Q13**	3.73	4	2	5	0.72	-0.22	-0.11
**Anxiety 1 –Q14**	2.73	3	1	5	1.08	-0.86	0.28
**Behavioral Intention 1 –Q15**	3.87	4	2	5	0.68	0.22	-0.29
**Performance Expectancy 2 –Q16**	3.85	4	2	5	0.69	0.47	-0.43
**Attitude Toward Using Technology 2 –Q17**	3.79	4	2	5	0.78	-0.1	-0.36
**Effort Expectancy 2 –Q18**	3.71	4	2	5	0.68	0.07	-0.24
**Anxiety 2 –Q19**	2.47	2	1	5	0.94	-0.38	0.67
**Performance Expectancy 3 –Q20**	3.28	3	1	5	0.83	0.19	-0.32
**Behavioral Intention 2 –Q21**	3.84	4	2	5	0.63	1.44	-0.73
**Anxiety 3 –Q22**	2.66	2	1	5	0.96	-0.86	0.25

**Fig 3:**
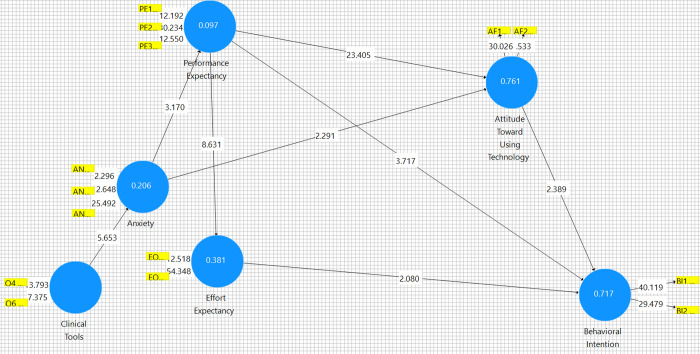
Final Path Model generated using SmartPLS v. 3.2.9 Bootstrapping.

Partial Least Squares (PLS) SEM was utilized to investigate the relationship between constructs. PLS-SEM calculations were performed using SmartPLS V. 3.2.9. Complete data analysis steps are included in the Supplemental Data Analysis (Section A.3 in S1 Appendix). SEM began with connecting all possible paths, then eliminating construct relationships that were insignificant. The final SEM is presented in Fig 3 and details in Table 3. T statistics for each path are greater than 1.95 and p values are below 0.05, indicating that each relationship is statistically significant.

**Table 3:** Final Path Coefficient Report generated using SmartPLS v. 3.2.9 Bootstrapping.

	Original Sample (O)	Sample Mean (M)	Standard Deviation (STDEV)	T Statistics (|O/STDEV|)	P Values
**Anxiety -> Attitude Toward Using Technology**	-0.127	-0.127	0.056	2.291	0.022
**Anxiety -> Performance Expectancy**	-0.312	-0.326	0.098	3.17	0.002
**Attitude Toward Using Technology -> Behavioral Intention**	0.329	0.326	0.138	2.389	0.017
**Clinical Tools -> Anxiety**	0.453	0.464	0.08	5.653	0
**Effort Expectancy -> Behavioral Intention**	0.202	0.194	0.097	2.08	0.038
**Performance Expectancy -> Attitude Toward Using Technology**	0.824	0.824	0.035	23.405	0
**Performance Expectancy -> Behavioral Intention**	0.399	0.408	0.107	3.717	0
**Performance Expectancy -> Effort Expectancy**	0.617	0.619	0.072	8.631	0

**Fig 4:**
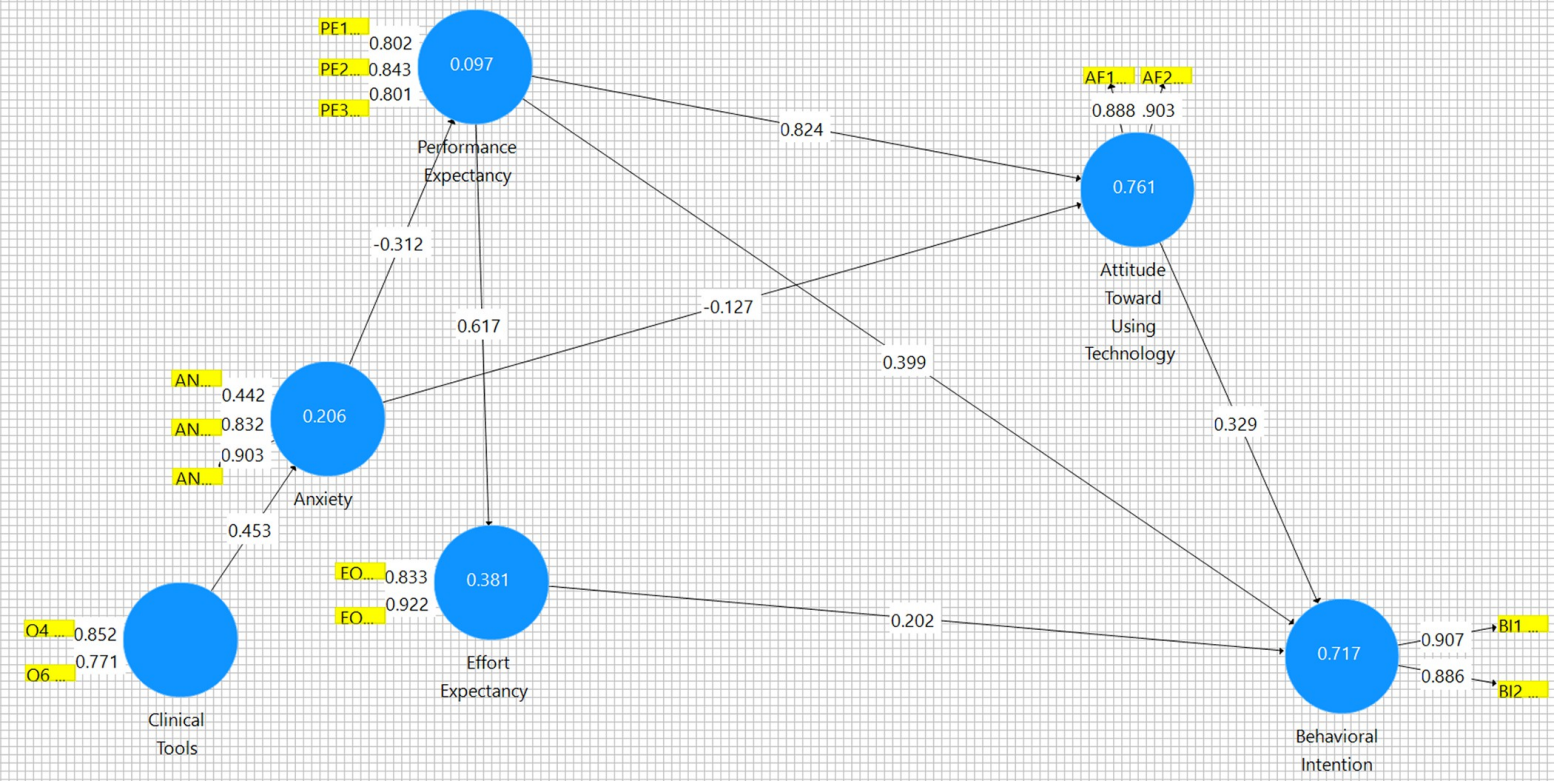
Partial Least Squares Path Model generated using SmartPLS v. 3.2.9 PLS.

Cronbach’s Alpha report (Table A.6 in S1 Appendix) shows that the t statistic is greater than double the standard deviation, and this indicates the model fits 95% of the data. Average Variance Extracted report (Table A.7 in S1 Appendix) and Construct Reliability and Validity report (Table A.8 in S1 Appendix) show strong model fit, reliability, and validity of remaining constructs. Fig 4 Partial Least Squares model was created to determine path coefficients–Table 4. These reports explain the model and variance encountered in the model. The weakest relationships surround anxiety. Based on this analysis, we know that Clinical Tools strongly influence anxiety, however, Clinical Tools has the lowest Cronbach’s Alpha and Adjusted Rho of all reviewed items. Anxiety also has a less than ideal Cronbach’s Alpha, but other indicators show that it is likely a reliable concept.

**Table 4:** Partial Least Squares Path Coefficients Report generated using SmartPLS v. 3.2.9 PLS.

	Anxiety	Attitude Toward Using Technology	Behavioral Intention	Clinical Tools	Effort Expectancy	Performance Expectancy
**Anxiety**		-0.127				-0.312
**Attitude Toward Using Technology**			0.329			
**Behavioral Intention**						
**Clinical Tools**	0.453					
**Effort Expectancy**			0.202			
**Performance Expectancy**		0.824	0.399		0.617	

### 3.2 Interview data analysis

**Fig 5:**
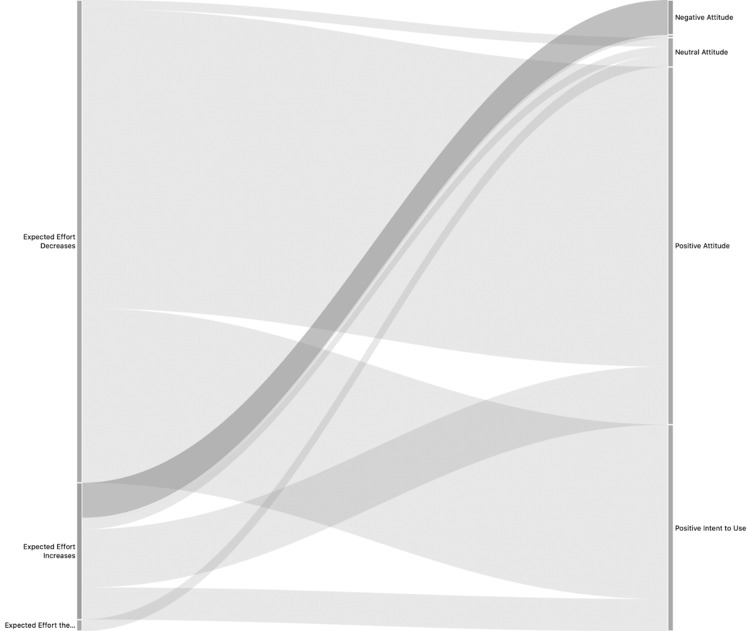
First level interactions Sankey diagram generated using ATLAS.ti 9.0.19.0.

The average interview time was 39.93 minutes. Krippendorff’s CU Alpha was generated at an individual narrative (Table B.3 in S1 Appendix) and overall level. Interviews were eliminated until the overall level reached α ≥ .8, resulting in 5 interviews eliminated and an overall α = 0.82. Code co-occurrence was measured by hypothesis and Sankey diagrams generated (Fig 5). Section B.4 in S1 Appendix includes hypothesis testing, interpretation, and Sankey diagrams (figures B.4.1–B.4.7) for each hypothesis.

### 3.3 Survey results

Table 5 includes the outcomes of each hypothesis for the survey. Results are expanded upon in Section A.4. in S1 Appendix. Hypotheses were tested at a 95% significance level.

**Table 5:** Survey Hypotheses tested, P-values, and outcomes.

Hypothesis	P-Value	Reject Null
**H1: Radiologists have intent to use an IM based conversational agent**	<0.001	Yes
H1-A: Intent is moderated by performance expectancies	<0.001	Yes
H1-B: Intent is moderated by effort expectancies	<0.001	Yes
H1-C: Intent is moderated by anxiety	0.514	No
H1-D: Intent is moderated by age	0.103	No
H1-E: Intent is moderated by radiologist’s experience with general consumer conversational agents and experience with radiology domain specific clinical tools	0.336	No
H1-F: Intent is moderated by attitude toward the system	0.017	Yes
**H2: Radiologists attitude toward the IM based conversational agent is positive**	<0.001	Yes
H2-A: Attitude is moderated by performance expectancies	<0.001	Yes
H2-B: Attitude is moderated by effort expectancies	0.056	No
H2-C: Attitude is moderated by anxiety	0.022	Yes
H2-D: Attitude is moderated by age	0.103	No
H2-E: Attitude is moderated by radiologist’s experience with general consumer conversational agents and experience with radiology domain specific clinical tools	0.371	No
**H3: Age influences radiologist’s perspective of the intervention**	Multiple	No
**H4: Experience with consumer conversational agent’s moderate’s radiologist’s perspective of the intervention**	Multiple	No
**H5: Experience with consumer and clinical IM tools moderate’s radiologist’s perspective of the intervention.**	Multiple	No
**H6: Experience with radiology domain specific clinical tools moderate’s radiologist’s perspective of the intervention.**	Multiple	Yes

### 3.4 Interview results

Table 6 includes the outcomes of each hypothesis for the interview. Interpretation, code co-occurrence tables, and Sankey diagrams supporting results are found in Section B.4 figures B.4.1-B.4.7 in S1 Appendix. Quotes related to common themes are included in Section B.5 in S1 Appendix.

**Table 6:** Interview Hypotheses tested.

Hypothesis	Reject Null
**H1: Expected effort is not a contributing factor in attitude towards the intervention but is a contributing factor in intent to use the intervention.**	No
**H2: With respect to this intervention, anxiety has a negative relationship with performance expectancy. As anxiety increases, performance expectancy decreases. As anxiety decreases, performance expectancy increases.**	No
**H3: Radiologists have a positive attitude towards this intervention and a high intent to use this intervention if it were produced.**	Yes
**H4: For this intervention, intent to use and attitude are mostly influenced by performance expectancies.**	No
H4-A: Radiologist’s attitude towards this intervention is mostly influenced by the expected performance of the system.	No
H4-B: Radiologist’s intent to use this intervention is mostly influenced by expected performance of the system.	No
**H5: Radiologist’s performance expectancies positively influence their effort expectancies. As expected performance increases, expected effort decreases.**	Yes

## 4 Discussion

Radiologists have a high intent to use and positive attitudes towards IM based CDSS and the presented interventions overall. We determined that years of experience, and Consumer Tools (IM and CA) were not moderating variables in our model. In any given path, the t statistic was too low and p value too high to consider this in our analysis. These questions are not a part of the UTAUT model, and we found them not to be factors relevant to our efforts. The following UTAUT expected paths were additionally removed, and speculation as to why is included.

### 4.1 Age and intent to use

This is a deviation from UTAUT which expects younger users to be more accepting of new software than older users. Potentially, radiologists are technologically saturated users; they perform their work functions using a wide variety of complex technological solutions. Among clinicians, radiologists chose this specialty because of their interest in technology solutions within healthcare. We were unable to measure this result during interviews.

### 4.2 Expected efforts influence on attitude

The survey and interview studies have differing results for expected efforts influence on attitude. The survey deviated from UTAUT in not finding a statistically significant association between effort and attitude. However, the interview study showed that decreasing effort is linked to positive attitude and positive intent to use, which is what we would expect in any technology usability study. Potentially the single example given in the survey was not enough to reveal this connection. Common themes on effort/attitude interactions

- Reducing time to acquire and apply clinical knowledge. The task of looking up non-imaging data within the EMR or clinical guidelines adds up quickly. CDSS’s ability to reduce this time is valued.
- Increasing the ability to multitask by enhancing images with useful information such as patient risk factors that are related to the exam being read.
- “Trusting CDSS as safety nets that ensure every necessary step of the workflow is automated or confirmed, for example incidental finding or critical results communications.

### 4.3 Anxieties influence on attitude

The survey shows a small negative relationship with attitude, which is the expected path in UTAUT. As anxiety increases, the user’s attitude toward using the technology decreases. The interview study asked many questions to understand anxiety surrounding this intervention, however, we were unable to strongly correlate with attitude. Overall, anxiety is the least grounded concept throughout the interview.

### 4.4 Expected performance as the major influencer of attitude and intent to use

Overall, expected performance is a major influence on attitude and intent to use. Within the survey results it has significantly more influence than any other factor. However, the interview results show a stronger correlation of expected effort with attitude and intent to use. There is a strong negative relationship between performance and effort present in both phases of the study, another deviation from the UTAUT model. There is potential that radiologists’ system use is derived from performance, maybe measured in clinical outcomes. However, we cannot assume these performance metrics overcome effort needs. Common themes from factors influencing attitude/intent to use-

- Radiologists expect to be interrupted or context switch quickly. CDSS tools for radiologists can leverage this expectation, but there is a lot of room to reduce mental load in simple tasks such as worklist management.
- Reducing effort is highly embraced. Tools that automate routine workflow steps such as looking up clinical guidelines or communicating with providers or staff are spoken of frequently.
- Radiologists will trade effort for performance. Even if they have to parse more information, if that information is relevant to the study they are reading they find it useful. Interventions that lead to higher reimbursement rates are accepted even if they require more effort. Decreasing overall productivity can be acceptable if you also increase the quality of their work.

### 4.5 Limitations

The survey data was collected using convenience sampling at one academic health care institution. Future studies could be done sampling radiologists from a broader audience.

While CA are in vogue now, this study was completed in 2019 and 2020. This was a novel design for a CDSS tool. This study is proof of concept and further development and study is warranted. In light of recent advancements in the field surrounding LLM and generative AI models, the results of this study may be different.

UTAUT is designed to collect data on a system that exists and that the users can interact with. We have leveraged aspects of UTAUT to collect data on a system that could exist and performed SEM analysis to generate a new model.

### 4.6 Conclusion

Radiologist’s interactions with decision support tools, or at least this intervention, differs from the standard user software interaction model. The positive relationship from performance to effort is the most major deviation, allowing increasing effort if the outcomes are desirable enough. This relationship is supported by both the survey and interview studies. Further, because performance and effort make up most of attitude and intent to use, there are a lot of opportunities for CDSS to provide novel workflow changes that increase patient outcomes. CDSS should be designed to streamline activities, and we see particular interest in tools to enable clinical knowledge gathering and context switching.

Anxiety is another deviation from the standard user model. In both parts of the study anxiety had the weakest relationships and was often secondary to the excitement of new clinical solutions. The most common source of anxiety surrounds the maintenance of CDSS. This suggests that radiologists are users with low technological anxiety compared to the general population and that they may be more accepting of advancements in their tools. This is reflected in radiology’s transformation from analog to digital over the last 50 years [1–4].

Radiologists deviate from the standard clinician with regards to the 10 commandments of CDSS. Commandments 2, 3, 7, 10, and 1 –anticipate needs, fit into user workflow, simple interventions, knowledge system maintenance, and speed–are all highlighted within radiology specific guidance, and we do find these present for radiologists in our study. However, the relationship between performance and effort highlights that radiologist CDSS doesn’t need to always hit every commandment. Radiologists expect workflow modification, they routinely use complex interventions, and they are not overwhelmed by CDSS information gathering. As we design for the future radiologist, we can trade effort in these commandments for increasing positive outcomes.

## Supporting information

S1 Appendix Detailed information on the study including the full survey, expanded hypothesis results, Semi-structured interview guide, code co-occurrence tables, full data analysis/findings, and additional diagrams. (DOCX)

S1 Data Raw quantitative survey data in Excel format. (CSV)
