# Supplementary material for: Theory of radiologist interaction with instant messaging decision support tools: A sequential-explanatory study
Source: PLOS Digit Health. 2024 Feb 26;3(2):e0000297. doi: 10.1371/journal.pdig.0000297 (PMC10896537; doi:10.1371/journal.pdig.0000297)
Supplement: S1 Appendix — (DOCX) [file pdig.0000297.s001.docx]

# **Appendix**

# **A.1 Survey In Full**

In developing the survey, we chose at least 2 questions for each construct/factor (denoted in bold, but not revealed in the survey) with slight modifications (denoted in italics) focusing users on the intervention in question. Questions were arranged so that no similar factors question was directly before or after another. The survey is designed to be completed in less than 10 minutes and respects the radiologist expertise in systems they utilize regularly in routine patient care. During development, the survey was tested by 2 undergraduate students studying programming and one radiology scheduling professional. These users averaged 5.66 minutes to survey completion.

Below is the survey in full. Response options are radio buttons of a 5 point Likert scale, ranging from 1 to 5 disagree to agree, unless otherwise noted [76]. The use case is based on a LUNG-RAD structured report found in the Capturing Data Elements and the Role of Imaging Informatics presented by Dr. William Hsu [72].

Instant Messaging CDSS Intervention Feasibility and Usability Quantitative Survey

Welcome and thank you for agreeing to complete our survey. Responses are anonymous and analysis of data will be aggregated before review. No responses will be published at an individual level.

There are 4 sections of this survey, on 4 pages, designed to collect:

1. Your consent to participate in this study.
2. Background information about your experience.
3. Demonstrate one use case of automated instant messaging as a decision support tool. Collect your feedback on this use case.
4. A. Describe additional use cases.
5. B. Collect your feedback on the overall concept of automated instant messaging communications for clinical decision support systems.

Acronyms utilized throughout this survey include: Clinical Decision Support (CDS) Picture Archiving and Communication Systems (PACS); Radiology Information Systems (RIS); Voice Recognition (VR); Instant Message (IM).

#### Section 1: Consent

**PARTICIPANT’S CONSENT**

I reviewed the study information that was emailed to me. Based on that I give my consent to participate in this research study. By continuing, I agree to take part in this anonymous study.

#### Section 2: User Background Information

1. How many years approximately have you practiced medicine as a radiologist?
   - 0-5; 5-10; 10-15; 15-20; 20+
2. Approximately how many years old are you?
   - 20-30; 31-40; 41-50; 51-60; 61-70; 70+
3. What is your subspecialty?
   - Fill in the blank
4. How difficult do you find PACS/RIS/VR systems to use?
   - Ranging from 1 to 5; 1 - Easy to use; 3 - Neutral; 5 - Difficult to use
5. How difficult do you find IM tools (IE WhatsApp, Facebook Messenger, hangouts, text messaging, AOL IM) to use?
   - Ranging from 1 to 5; 1 - Easy to use; 3 - Neutral; 5 - Difficult to use
6. How difficult do you find clinical IM tools to use?
   - Ranging from 1 to 5; 1 - Easy to use; 3 - Neutral; 5 - Difficult to use
7. How difficult do you find conversation agents (IE Siri, Alexa, Google Assistant, internet store/technology agents) to use?
   - Ranging from 1 to 5; 1 - Easy to use; 3 - Neutral; 5 - Difficult to use

#### Section 3: Clinical Decision Support Using Automated Instant Messaging Use Case

The intervention below assumes that our tool has access to data in the patient record including PACS. In this case, the tool is reviewing a draft version of your report using natural language processing as well as metadata surrounding the examination (such as procedure code, dose information, and history) The IM you receive suggests an addition to the report, including a formatted LUNG-RAD score and follow-up recommendation based on clinical guidelines. The example report, criteria for formulating LUNG-RAD scores, and output text is pulled from [Capturing Data Elements and the Role of Imaging Informatics](http://amos3.aapm.org/abstracts/pdf/99-27434-359478-111844-1383861762.pdf) presented by Dr. William Hsu found on slide 13 Structured Reporting (review of this document is not required, but provided for background).

Assume that the language of the intervention is based on institution specific implementations and approved through a CDS governance board. If LUNG-RAD scoring is not relevant to your work, imagine the following scenario with another scoring tool that is more relevant (IE BI-RAD, PI-RAD, C-RAD, RECIST, etc.).

After completing the draft report, you receive an IM notification. The notification’s appearance is shown below as the orange highlight surrounding the IM tool on a PACS workstation. This conversation initiates two seconds after saving the draft report.


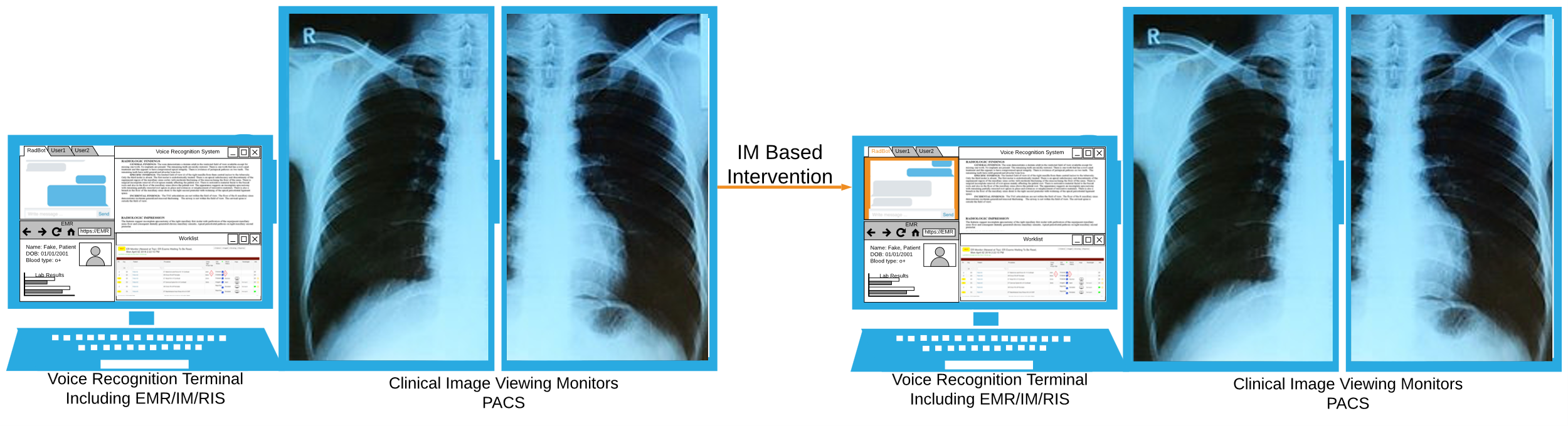


Figure A.1.1 Sample PACS workstation before/after IM based intervention. Source images for Lung X-Ray [69], Report [70], and IM transaction [71].

Text of the IM conversation follows. Message authors are indicated by System for automated messages and Radiologist for your response.

System: Based on your report text I am initiating the LUNG-RADS reporting tool. I have 2 recommended additions. Type ‘Y’ to add to the report and ‘N’ to move on.

System: Addition after TECHNIQUE, before FINDINGS sections:
“RADIATION DOSE: 1 Volumetric series was performed for this exam.
CTDIvol (CT dose Index-volume) = 2.5 mG
DLP (Dose Length Product) = 80 mG cm”

Radiologist: Y

System: Addition in IMPRESSION:

“Lung Cancer Screening: LungRADS Category 2, Benign appearing (non-actionable) nodule(s). These types of nodules are commonly observed and require no immediate action. Current recommendations for eligible high risk individuals (criteria below) are routine annual screening with low dose CT.”

Radiologist: Y

System: Additions were added to the draft report and are now available for your review.

#### Section 3: Survey

The following questions use a Likert scale of -2 strongly disagree to +2 strongly agree, with a 0 indicating neutrality. Please answer all questions.

1. This alert was presented as a
   - Pop-up window; An email; My human assistant messaged me; A bot instant messaged me
2. How did the system generate the lung-rad score?
   - I filled in a form in an external website and it created the lung-rad score; It used natural language processing to understand my report and generate a LUNG-RAD score; It didn’t generate a LUNG-RAD score
3. What did the system do with the LUNG-RAD score once it was generated?
   - The LUNG-RAD score and guideline recommended follow-up was inserted into the report; Only the numerical LUNG-RAD score was inserted into the report; The LUNG-RAD score was presented to me and I could choose to copy-paste it into the report; The system did not generate a LUNG-RAD score.
4. **PE –** *Using this tool* enables me to accomplish tasks more quickly.
5. **EOU** **–** My interaction with the system would be clear and understandable.
6. **AF –** Using the system is a good idea.
7. **ANX –** It scares me to think that I could lose a lot of information using the system by hitting the wrong key.
8. **BI –** *If available, I would* use this system.

#### Section 4: Clinical Decision Support Using Automated Instant Messaging Overall

With an understanding of one use case, hopefully you are imagining many other workflows such a tool could augment. Other use cases we are proposing include:

- Guideline recommended care additions based on findings
  - “Given findings you presented this is the latest ACR guidelines…Would you like to update impression to include these?”
- Workflow Management and prioritization
  - “You are ahead on your worklist, Dr. Smith’s list currently includes a priority 0 stroke case and 3 priority 1 cases. Would you like to open this worklist in PACS?”
  - “We found another completed exam for the patient you just finalized of PROCEDURE CODE X. Would you like to open this exam?”
- Billable report text corrections
  - “Was this exam processed with the 3D reconstruction tool? Type ‘Y’ to include in report”
- Motivational messages
  - “You are currently exceeding average RVU output of this seat, keep up the great work!”

#### Section 4: Overall Survey

Now that you have an understanding of possible implementations of the tool, we would like to ask questions about the concept in general. The following questions are to be answered about IM based CDS in general, and are not addressing any individual intervention.

The following questions use a Likert scale of -2 strongly disagree to +2 strongly agree, with a 0 indicating neutrality. Please answer all questions.

1. **PE -** I would find the system useful in my job.
2. **AF –** I *would* like *to work* with the system.
3. **EOU –** I would find *IM based CDS* easy to use.
4. **ANX –** The system is somewhat intimidating to me.
5. **PE -** Using the system increases my productivity.
6. **BI –** I predict I *could* use the system *as part of routine diagnostic work.*
7. **ANX –** I feel apprehensive about using the system.

#### Section 5: Concluding Remarks

Thank you for your time, interest, and support of this project. This concludes the quantitative data collection steps. If you would like to participate in the qualitative interview and have not yet indicated such, please email [jolburns@iupui.edu](mailto:jolburns@iupui.edu) for more information.

# Table A.2 Survey questions with bucketing

| Question | Text | UTAUT Concept | Bucketing |
| --- | --- | --- | --- |
| 1 | How many years approximately have you practiced medicine as a radiologist? |  | 1: 0-5; 2: 5-10; 3: 10-15; 4: 15-20; 5: 20+ |
| 2 | Approximately how many years old are you? |  | 1: 20-30; 2: 31-40; 3: 41-50; 4: 51-60; 5: 61-70; 6: 70+ |
| 3 | What is your subspecialty? |  | Converted free text responses into formatted result (IE “Resident, no subspecialty” and “Diagnostic Radiology Resident” become “resident” |
| 4 | How difficult do you find PACS/RIS/VR systems to use? |  | Likert 1-5 |
| 5 | How difficult do you find IM tools (IE WhatsApp, Facebook Messenger, hangouts, text messaging, AOL IM) to use? |  | Likert 1-5 |
| 6 | How difficult do you find clinical IM tools to use? |  | Likert 1-5 |
| 7 | How difficult do you find conversation agents (IE Siri, Alexa, Google Assistant, internet store/technology agents) to use? |  | Likert 1-5 |
| 8 | This alert was presented as a |  | A: Pop-up window; B: An email; C: My human assistant messaged me; D: A bot instant messaged me |
| 9 | How did the system generate the lung-rad score? |  | A: I filled in a form in an external website and it created the lung-rad score; B: It used natural language processing to understand my report and generate a LUNG-RAD score; C: It didn’t generate a LUNG-RAD score |
| 10 | What did the system do with the LUNG-RAD score once it was generated? |  | A: The LUNG-RAD score and guideline recommended follow-up was inserted into the report; B: Only the numerical LUNG-RAD score was inserted into the report; C: The LUNG-RAD score was presented to me and I could choose to copy-paste it into the report; D: The system did not generate a LUNG-RAD score. |
| 11 | *Using this tool* enables me to accomplish tasks more quickly. | PE1 | Likert 1-5 |
| 12 | My interaction with the system would be clear and understandable | EOU1 | Likert 1-5 |
| 13 | Using the system is a good idea. | AF1 | Likert 1-5 |
| 14 | It scares me to think that I could lose a lot of information using the system by hitting the wrong key. | ANX1 | Likert 1-5 |
| 15 | *If available, I would* use this system. | BI1 | Likert 1-5 |
| 16 | I would find the system useful in my job. | PE2 | Likert 1-5 |
| 17 | I *would* like *to work* with the system. | AF2 | Likert 1-5 |
| 18 | I would find *IM based CDS* easy to use. | EOU2 | Likert 1-5 |
| 19 | The system is somewhat intimidating to me. | ANX2 | Likert 1-5 |
| 20 | Using the system increases my productivity. | PE3 | Likert 1-5 |
| 21 | I predict I *could* use the system *as part of routine diagnostic work.* | BI2 | Likert 1-5 |
| 22 | I feel apprehensive about using the system. | ANX3 | Likert 1-5 |

# A.3 Supplemental Data Analysis

## Table A.3.1 SmartPLS Bootstrapping Configurations

| Data file Settings | |
| --- | --- |
| Data file | Survey Data for Analysis [86 records] |
| Missing value marker | none |
| Data Setup Settings | |
| Algorithm to handle missing data | None |
| Weighting Vector | - |
| PLS Algorithm Settings | |
| Data metric | Mean 0, Var 1 |
| Initial Weights | 1 |
| Max. number of iterations | 2000 |
| Stop criterion | 7 |
| Use Lohmoeller settings? | No |
| Weighting scheme | Path |
| Bootstrapping Settings | |
| Complexity | Complete Bootstrapping |
| Confidence interval method | Bias-Corrected and Accelerated (BCa) Bootstrap |
| Parallel processing | Yes |
| Samples | 5000 |
| Significance level | 0.05 |
| Test type | Two Tailed |
| Construct Outer Weighting Mode Settings | |
| Anxiety | Automatic |
| Attitude Toward Using Technology | Automatic |
| Behavioral Intention | Automatic |
| Clinical Tools | Automatic |
| Effort Expectancy | Automatic |
| Performance Expectancy | Automatic |

##

## Table A.3.2 SmartPLS Partial Least Squares Configuration

| Data file Settings | |
| --- | --- |
| Data file | Survey Data for Analysis [86 records] |
| Missing value marker | none |
| Data Setup Settings | |
| Algorithm to handle missing data | None |
| Weighting Vector | - |
| PLS Algorithm Settings | |
| Data metric | Mean 0, Var 1 |
| Initial Weights | 1 |
| Max. number of iterations | 5000 |
| Stop criterion | 7 |
| Use Lohmoeller settings? | No |
| Weighting scheme | Path |
| Construct Outer Weighting Mode Settings | |
| Anxiety | Automatic |
| Attitude Toward Using Technology | Automatic |
| Behavioral Intention | Automatic |
| Clinical Tools | Automatic |
| Effort Expectancy | Automatic |
| Performance Expectancy | Automatic |

## Structured Equation Modeling Analysis

Partial Least Squares (PLS) Structured Equation Modeling (SEM) was utilized to investigate the relationship between constructs. “SEM is a considerably complex statistical method for assessing relations between constructs, including latent and observed variables. Latent variables refer to the conceptual terms that are employed to show the theoretical concepts. These variables in the model are graphically symbolized by a circle. Observed variables refer to items, measures and indicators of variables that are measured directly and graphically represented by a square in the model…The estimation of a model delivers empirical measures of: 1) the relationships between the indicators and the constructs (measurement models or outer model) and 2) the relationships between the constructs (structural model or inner model) (Byrne, 2013b; Hair et al., 2014). The empirical measures allow the researcher to compare the theoretically established measurement and structural models with reality, as represented by the sample data. In other words, the empirical measures enable the researcher to determine how well the theory fits the data. Therefore, using PLS-SEM enabled the researcher to measure the model's predictive potential and competences to judge the quality of the model (Hair et al., 2014). (1)” PLS-SEM calculations were performed using SmartPLS V. 3.2.9 with settings listed in Tables A.3.1 and A.3.2. Figure A.3.1 reveals the expected relationship based on the UTAUT framework.


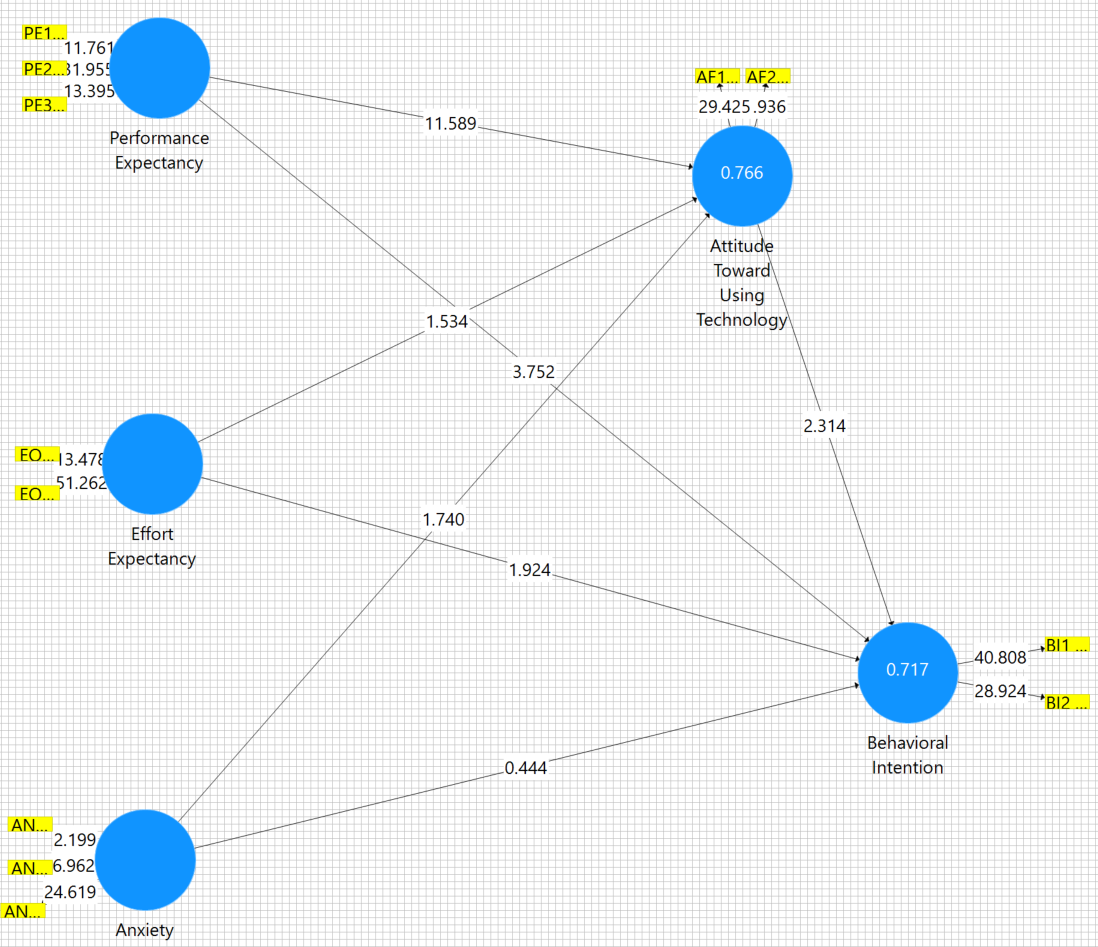


Figure A.3.1 UTAUT construct Path Model, generated using SmartPLS v. 3.2.9 Bootstrapping

From Figure A.3.1’s model we can infer based on path weights over 1.95 the significance of the path relationship. The strongest relationship is PE -> AF with 11.589 weight. Additionally, this model shows clear relationships between AF->BI, PE->BI. Potential weak relationships between EOU->BI and ANX->AF. The relationship ANX->BI is not very highly weighted. Initial conclusions from this model are that, for radiologists, BI and AF toward instant messaging conversational agents are driven mostly by PE.

|  | Original Sample (O) | Sample Mean (M) | Standard Deviation (STDEV) | T Statistics (\|O/STDEV\|) | P Values |
| --- | --- | --- | --- | --- | --- |
| AF1 - Q13 <- Attitude Toward Using Technology | 0.887 | 0.885 | 0.03 | 29.425 | 0 |
| AF2 - Q17 <- Attitude Toward Using Technology | 0.903 | 0.903 | 0.021 | 43.936 | 0 |
| ANX1 - Q14 <- Anxiety | 0.484 | 0.463 | 0.22 | 2.199 | 0.028 |
| ANX2 - Q19 <- Anxiety | 0.77 | 0.743 | 0.111 | 6.962 | 0 |
| ANX3 - Q22 <- Anxiety | 0.93 | 0.921 | 0.038 | 24.619 | 0 |
| BI1 - Q15 <- Behavioral Intention | 0.907 | 0.906 | 0.022 | 40.808 | 0 |
| BI2 - Q21 <- Behavioral Intention | 0.886 | 0.885 | 0.031 | 28.924 | 0 |
| EOU1 - Q12 <- Effort Expectancy | 0.843 | 0.832 | 0.063 | 13.478 | 0 |
| EOU2 - Q18 <- Effort Expectancy | 0.915 | 0.918 | 0.018 | 51.262 | 0 |
| PE1 - Q11 <- Performance Expectancy | 0.798 | 0.794 | 0.068 | 11.761 | 0 |
| PE2 - Q16 <- Performance Expectancy | 0.842 | 0.846 | 0.026 | 31.955 | 0 |
| PE3 - Q20 <- Performance Expectancy | 0.808 | 0.803 | 0.06 | 13.395 | 0 |

Table A.3.3 UTAUT construct Path Model Outer Loadings Report, generated using SmartPLS v. 3.2.9 Bootstrapping

Table A.3.3 details the outer loadings report from SmartPLS. The T-Statistic column is represented in Figure A.3.1 as the ‘loadings’ for the path between concepts. We present this to detail that all questions had an acceptable t-statistic and p value. We did not eliminate any concepts based on these factors.

We then bring in our moderating variables and link to the UTAUT concepts (Figure A.3.2). These are the concepts we expected to influence AF and BI – age and experience (questions 1,2); clinical tool experience (questions 4,6); consumer tool experience (questions 5,6).


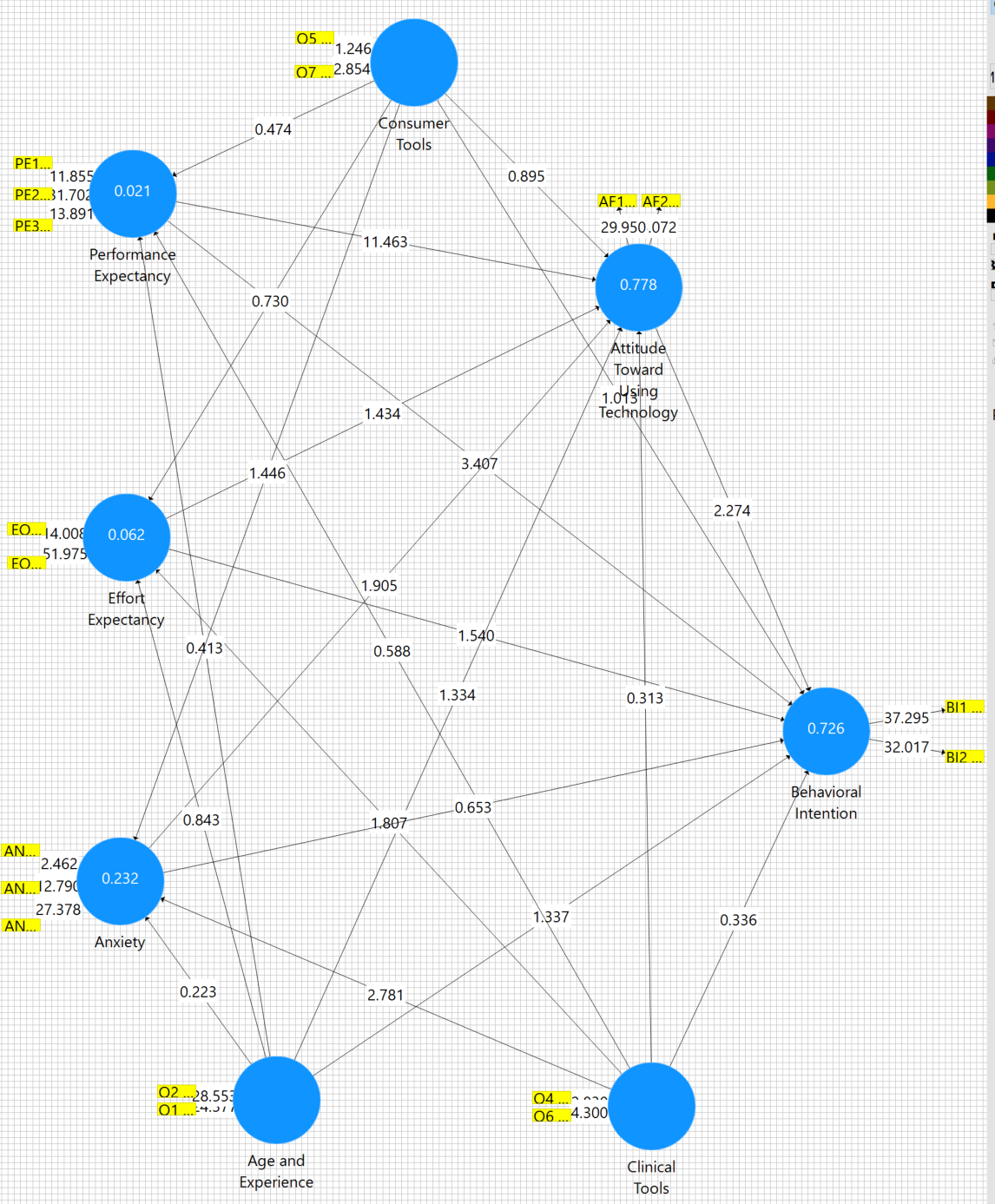


Figure A.3.2 UTAUT construct and moderating factors Path Model, generated using SmartPLS v. 3.2.9 Bootstrapping

As we add more paths, Figure A.3.2 SEM becomes more cluttered and harder to read. Note the difference in weight between PE->AF has lowered while the ANX->BI has increased. This difference is due to Bootstrapping, and minor variances are expected for each run. Table A.3.4 is a chart showing all paths, the path loading – t statistic, and the p value.

|  | T Statistics (\|O/STDEV\|) | P Values |
| --- | --- | --- |
| Age and Experience -> Anxiety | 0.223 | 0.824 |
| Age and Experience -> Attitude Toward Using Technology | 1.334 | 0.182 |
| Age and Experience -> Behavioral Intention | 1.337 | 0.181 |
| Age and Experience -> Effort Expectancy | 0.843 | 0.399 |
| Age and Experience -> Performance Expectancy | 0.413 | 0.68 |
| Anxiety -> Attitude Toward Using Technology | 1.905 | 0.057 |
| Anxiety -> Behavioral Intention | 0.653 | 0.514 |
| Attitude Toward Using Technology -> Behavioral Intention | 2.274 | 0.023 |
| Clinical Tools -> Anxiety | 2.781 | 0.005 |
| Clinical Tools -> Attitude Toward Using Technology | 0.313 | 0.754 |
| Clinical Tools -> Behavioral Intention | 0.336 | 0.737 |
| Clinical Tools -> Effort Expectancy | 1.807 | 0.071 |
| Clinical Tools -> Performance Expectancy | 0.588 | 0.557 |
| Consumer Tools -> Anxiety | 1.446 | 0.148 |
| Consumer Tools -> Attitude Toward Using Technology | 0.895 | 0.371 |
| Consumer Tools -> Behavioral Intention | 1.013 | 0.311 |
| Consumer Tools -> Effort Expectancy | 0.73 | 0.465 |
| Consumer Tools -> Performance Expectancy | 0.474 | 0.636 |
| Effort Expectancy -> Attitude Toward Using Technology | 1.434 | 0.152 |
| Effort Expectancy -> Behavioral Intention | 1.54 | 0.124 |
| Performance Expectancy -> Attitude Toward Using Technology | 11.463 | 0 |
| Performance Expectancy -> Behavioral Intention | 3.407 | 0.001 |

Table A.3.4 UTAUT construct and moderating factors Path coefficients, generated using SmartPLS v. 3.2.9 Bootstrapping

Path loadings are sorted to find the lowest loading, and then the smallest path is removed prior to rerunning the bootstrapping. We repeat this process until no paths remain with a t statistic less than 1.96 and a p value greater than .05, per the hypothesis criteria. Finally, we check alternate paths outside of the standard UTAUT model for relevant loadings; PE->EF, PE->ANX, ANX->EF, ANX->PE, EF->PE, EF->ANX. We found that each relationship had a t statistic greater than 1.95 and was significant. We kept ANX->PE and PE->EF as these were the strongest connections. This change is shown in figure A.3.3.


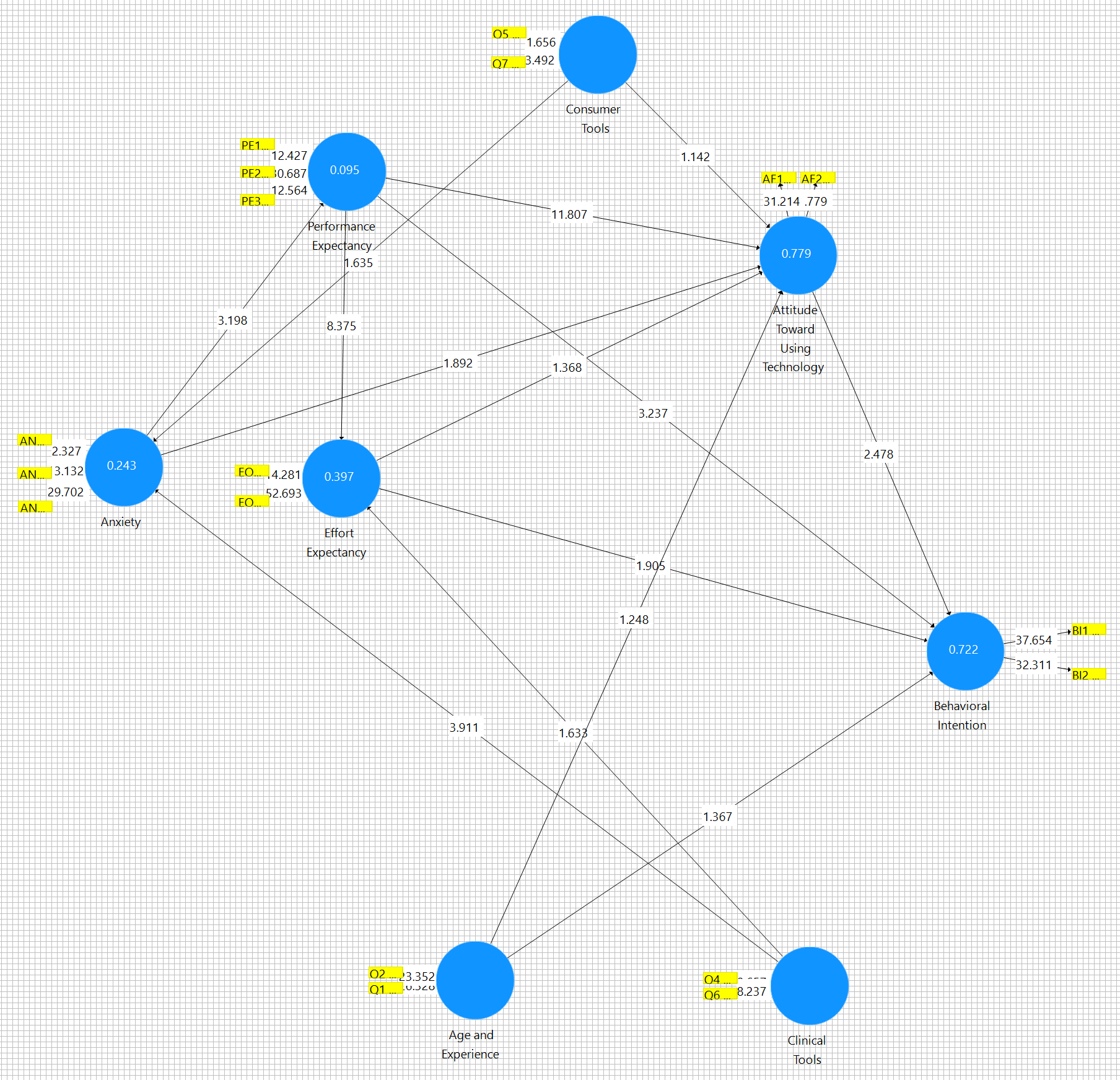


Figure A.3.3 UTAUT construct and moderating factors path coefficients modification outcomes Path Model, generated using SmartPLS v. 3.2.9 Bootstrapping

We then began trimming connections and observing the outcome on the path model. This helps explain the moderating factors. IE. Consumer Tools affects AF (1.142) and ANX (1.635), ANX affects AF (1.892); does removing the Consumer Tools->AF path strengthen the Consumer Tools->ANX relationship – yes, it improves the loading (1.739). This implies that the moderating factor Consumer Tools better explains the model through ANX than AF. During this exploration, we determined that the combination ‘Age and Experience’ factors was limiting their utility. The questions were split into distinct factors for analysis, then mapped to each UTAUT concept and reviewed. The strongest connections are Age->BI and Experience->AF. Figure A.3.4 is the diagram remaining after this effort.


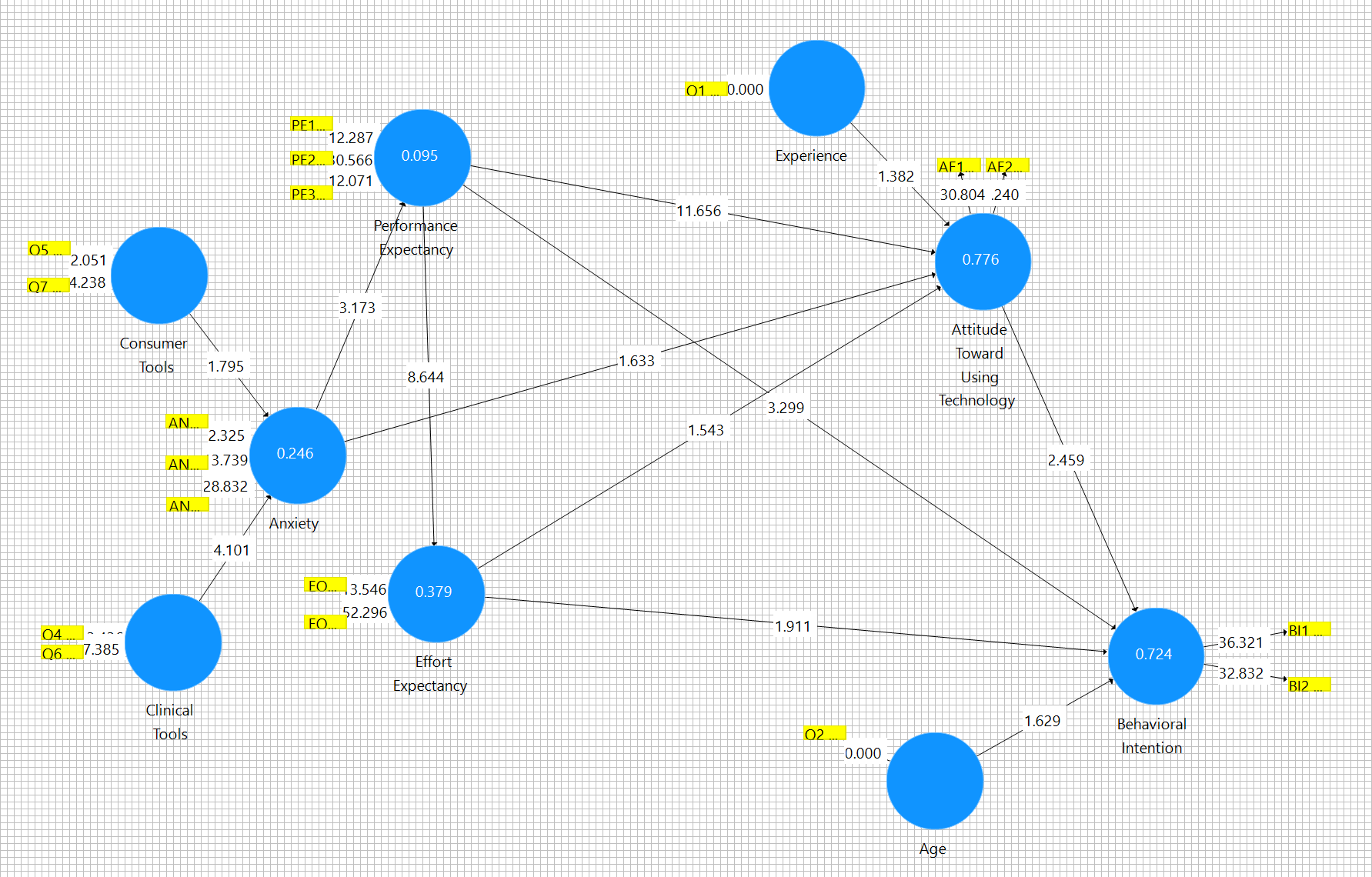


Figure A.3.4 UTAUT construct and moderating factors Path Model after reduction, generated using SmartPLS v. 3.2.9 Bootstrapping

|  | Original Sample (O) | Sample Mean (M) | Standard Deviation (STDEV) | T Statistics (\|O/STDEV\|) | P Values |
| --- | --- | --- | --- | --- | --- |
| Experience -> Attitude Toward Using Technology | 0.086 | 0.085 | 0.062 | 1.382 | 0.167 |
| Effort Expectancy -> Attitude Toward Using Technology | 0.128 | 0.124 | 0.083 | 1.543 | 0.123 |
| Age -> Behavioral Intention | -0.095 | -0.097 | 0.058 | 1.629 | 0.103 |
| Anxiety -> Attitude Toward Using Technology | -0.099 | -0.098 | 0.061 | 1.633 | 0.103 |
| Consumer Tools -> Anxiety | 0.208 | 0.231 | 0.116 | 1.795 | 0.073 |
| Effort Expectancy -> Behavioral Intention | 0.188 | 0.185 | 0.099 | 1.911 | 0.056 |
| Attitude Toward Using Technology -> Behavioral Intention | 0.356 | 0.347 | 0.145 | 2.459 | 0.014 |
| Anxiety -> Performance Expectancy | -0.308 | -0.323 | 0.097 | 3.173 | 0.002 |
| Performance Expectancy -> Behavioral Intention | 0.384 | 0.394 | 0.116 | 3.299 | 0.001 |
| Clinical Tools -> Anxiety | 0.387 | 0.387 | 0.094 | 4.101 | 0 |
| Performance Expectancy -> Attitude Toward Using Technology | 0.759 | 0.761 | 0.065 | 11.656 | 0 |
| Performance Expectancy -> Effort Expectancy | 0.615 | 0.618 | 0.071 | 8.644 | 0 |

Figure A.3.5 UTAUT construct and moderating factors Path Coefficients Report after reduction, generated using SmartPLS v. 3.2.9 Bootstrapping

From Figure A.3.4 and table A.3.5 data, we determined that Years of Experience as a radiologist and Consumer Tools (IM and CA) were not moderating variables in our model. In any given path, the t statistic was too low and p value too high to consider this in our analysis. Both were removed from further models. As we removed paths, bootstrapping is rerun, and the weakest paths are removed. In all we removed the paths Age->BI, EF->AF, and EF-AF.

# A.4 Survey Hypothesis Results

**H1: Radiologists have intent to use an IM based conversational agent**

Of the 2 BI questions asked, they hold an overall mean 3.855 and median 4, with low standard deviations (Table 2). The BI concept holds acceptable reliability and validity (Table 7) and a P-value <0.001 (Table 5). Based on this data we can reject the null hypothesis with 95% accuracy. Radiologists have a high intent to use an IM CA.

**H1-A: Intent is moderated by performance expectancies**

Of concepts in the final analysis, PE is the strongest moderator of BI, with a path weight of 0.399. The 3 PE questions have a mean 3.620 and median 3.667, with low standard deviations (Figure 3). PE has high reliability and validity per SEM analysis (Table 9) and the relationship has a P-value <0.001 (Figure 4). Based on this data we reject the null hypothesis. PE has a positive relationship with BI.

**H1-B: Intent is moderated by effort expectancies**

Of concepts in the final analysis, EOU is the weakest moderator of BI, with a path weight of 0.202. The 2 EOU questions have a mean 3.814 and median 4, with low standard deviations (Table 2). EOU has high reliability and validity per SEM analysis (Table 7) and the relationship has a P-value <0.001 (Table 3). Based on this data we reject the null hypothesis. Even though it is the weakest moderator, it is still significant. EOU has a positive relationship with BI.

**H1-C: Intent is moderated by anxiety**

ANX was not found to have a significant relationship with BI, with a P-value of 0.514 (Supplemental Data Analysis figure 6) and was not included in the final model. We are not able to reject the null hypothesis.

**H1-D: Intent is moderated by age**

Age was not found to be a significant moderator of any variable, including BI with a P-value 0.103 (Supplemental Data Analysis figure 9). We are not able to reject the null hypothesis.

**H1-E: Intent is moderated by radiologist’s experience with general consumer conversational agents and experience with radiology domain specific clinical tools**

Experience with clinical tools was not found to be a significant moderator of any variable, including BI with a P-value of 0.336 (Supplemental Data Analysis figure 6). We are not able to reject the null hypothesis.

**H1-F: Intent is moderated by their attitude toward the system**

Of concepts in the final analysis, AF is the middling moderator of BI, with a path weight of 0.329. The 2 AF questions have a high mean 3.762 and median 4, with low standard deviations (Table 2). AF has high reliability and validity per SEM analysis (Table 7) and the relationship has the P-value 0.017 (Table 3). Based on this data we reject the null hypothesis. AF is a significant moderator of BI. AF has a positive relationship with BI.

**H2: Radiologists attitude toward the IM based conversational agent is positive**

Based on the survey results, radiologists have a positive attitude toward IM CA for completing CDSS tasks. Of the 2 AF questions asked, they hold an overall mean 3.762 and median 4, with low standard deviations (Table 2) and has a P-value <0.001 (Table 5). Further, the SEM analysis results show the AF concept holds acceptable reliability and validity (Table 7). Based on this data we can reject the null hypothesis.

**H2-A: Attitude is moderated by performance expectancies**

Of concepts in the final analysis, PE is the strongest moderator of AF, with a path weight of 0.824. The 3 PE questions have a mean 3.620 and median 3.667, with low standard deviations (Table 2) and the relationship has a P-value <0.001 (Table 3). PE has high reliability and validity per SEM analysis (Table 7). Based on this data we reject the null hypothesis. PE has a strong positive relationship with AF.

**H2-B: Attitude is moderated by effort expectancies**

EOU was not found to have a significant relationship with AF, P-value of 0.056 (Supplemental Data Analysis figure 9) and was not included in the final model. We are not able to reject the null hypothesis.

**H2-C: Attitude is moderated by anxiety**

Of concepts in the final analysis, ANX is the very weak moderator of AF, with a path weight of negative 0.127. The 3 ANX questions have a mean 2.620 and median 2.333, with low standard deviations (Table 2). ANX did have a low Cronbach’s Alpha, but other indicators show high reliability and validity per SEM analysis (Table 7) and the relationship has a P-value 0.022 (Table 3). Based on this data we reject the null hypothesis. ANX has a weak negative relationship with AF.

**H2-D: Attitude is moderated by age**

Age was not found to be a significant moderator of any variable, including BI with a P-value of 0.103 (Supplemental Data Analysis figure 9). We are not able to reject the null hypothesis.

**H2-E: Attitude is moderated by radiologist’s experience with general consumer conversational agents and experience with radiology domain specific clinical tools**

Experience with consumer tools and clinical tools were not found to have a significant relationship with AF, P-value 0.371 (Supplemental Data Analysis figure 6) and was not included in the final model. We are not able to reject the null hypothesis.

**H3: Age influences radiologist’s perspective of the intervention**

Age has a mean 2.523 and median 2. Years practiced has a mean 2.326 and a median 2, with the largest standard deviation 1.434 of all measured questions. Neither was found to be a significant moderator of any variable. We are not able to reject the null hypothesis.

**H4: Experience with consumer conversational agents moderates radiologist’s perspective of the intervention**

Consumer CA has a mean 3.128 and a median 3. It was not found to be a significant moderator of any variable. We are not able to reject the null hypothesis.

**H5: Experience with consumer and clinical IM tools moderate’s radiologist’s perspective of the intervention**

Consumer IM tools has a mean 2.488 and median 2. Clinical IM tools has a mean 3.023 and a median 3. The most significant relationship was with ANX, with a P-value 0.073 (Supplemental Data Analysis figure 9). Consumer IM Tools was not found to be a significant moderator of any variable. We are not able to reject the null hypothesis.

**H6: Experience with radiology domain specific clinical tools moderate’s radiologist’s perspective of the intervention**

Experience with clinical tools was found to be a moderator of ANX, with a path weight of 0.453 and P-value <0.001 (Table 3). Clinical tools hold a low Cronbach’s Alpha and adjusted rho, with an acceptable composite reliability per SEM analysis (Table 7). It is a moderating factor for ANX, we can reject the null hypothesis.

# Table A.5 Interview research statements

| Research Statement | Reasoning | Interview Goal |
| --- | --- | --- |
| 0 - Radiologist training and expertise in technology eliminates age as a factor in technology acceptance. | UTAUT framework implies that age should be a moderating factor. Our survey findings have found that age is not a moderating factor for any path in our population model. The strongest path held a t statistic of 1.611 and p value of 0.107 and was removed from further survey analysis. | Interview a wide age range of radiologists and gain an understanding of an intent to use such a system. *Note – not able to be analyzed using code co-occurrence measurements and not reported.* |
| 1 – The expected effort in using our intervention does not change the attitude towards using the system, instead directly affecting the intent to use the system. | The UTAUT path EF->AF is not represented by our population sample. Instead we find that PE strongly influences EF, and that EF is only meaningfully affecting BI. EF->BI is a weak positive relationship. Radiologists expect performance to influence effort, and performance to be the main modifier surrounding intent to use and attitude towards the system. | If the system improved patient outcomes, what level of effort would a radiologist accept to accomplish this? How does this affect their attitude towards and intent to use our intervention? |
| 2 – Anxiety can be used as a predictor of expected performance of an automated IM CDS system. | In the final SEM bootstrapping and PLS models, the relationships surrounding ANX are the least reliable per some paths but not all. The UTAUT framework expects ANX to be a contributing factor in AF and BI. We did not find a strong link in these paths. We did find a UTAUT deviation, in the medium negative relationship ANX->PE. | Measure the radiologist’s existing anxiety surrounding IM CDS. |
| 3 – Radiologists have a positive attitude towards the automated IM CDS intervention and an intent to use it if it were produced. | AF->BI is a medium positive relationship. This is explained in UTAUT, in that if the user has a positive attitude of the technology, they will have a strong intent to use the technology. | Determine the radiologists attitude surrounding our intervention and their intent to use the system. |
| 4a – Radiologist’s attitude towards this intervention is mostly influenced by the expected performance of the system. | Radiologists measure performance in different ways, such as quality of patient care and report turn around time. UTAUT expects the PE->AF relationship. The strength of this relationship in our model is interesting. | Define what radiologists expect performance to be and determine their anticipated performance opportunities through use of this system. Analyze other factors influencing attitude and determine if performance is the main factor. |
| 4b – Radiologist’s intent to use this intervention is mostly influenced by performance of the system. | The UTAUT path, PE-BI, is validated by our survey data and is a medium positive relationship. | Analyze other factors influencing intent to use the system and determine if performance is the main factor. |
| 5 – Radiologist’s expectancies surrounding performance influence their expectancies surrounding effort. | This is not a standard UTAUT pathway. Survey analysis findings indicate PE->EF is a strong positive relationship. Radiologists expect their effort to be moderated by the overall performance of the system. This suggests that a poor performing system would decrease effort metrics, reducing attitude and intent to use, while a well performing system would increase effort and increase other metrics.  *Note: expected effort is measured opposite of what the reader may expect. Questions like “My interaction with the system would be clear and understandable” are used to measure effort. This means that an increase in expected effort is not an increase in the amount of effort to performs tasks in the system, instead an increase in the positive aspects of effort.* | Determine how performance and effort are linked by this group of experts. Analyze the limits of this relationship – what extra burdens would a radiologist accept to improve quality metrics. |

# Table A.6 Cronbach’s Alpha Report generated using SmartPLS v. 3.2.9 Bootstrapping

|  | Original Sample (O) | Sample Mean (M) | Standard Deviation (STDEV) | T Statistics (\|O/STDEV\|) | P Values |
| --- | --- | --- | --- | --- | --- |
| Anxiety | 0.617 | 0.61 | 0.085 | 7.298 | 0 |
| Attitude Toward Using Technology | 0.752 | 0.747 | 0.066 | 11.485 | 0 |
| Behavioral Intention | 0.756 | 0.752 | 0.068 | 11.075 | 0 |
| Clinical Tools | 0.489 | 0.484 | 0.131 | 3.734 | 0 |
| Effort Expectancy | 0.713 | 0.704 | 0.08 | 8.92 | 0 |
| Performance Expectancy | 0.755 | 0.751 | 0.054 | 13.97 | 0 |

# Table A.7 Average Variance Extracted (AVE) Report generated using SmartPLS v. 3.2.9 Bootstrapping

|  | Original Sample (O) | Sample Mean (M) | Standard Deviation (STDEV) | T Statistics (\|O/STDEV\|) | P Values |
| --- | --- | --- | --- | --- | --- |
| Anxiety | 0.568 | 0.569 | 0.051 | 11.249 | 0 |
| Attitude Toward Using Technology | 0.801 | 0.8 | 0.041 | 19.594 | 0 |
| Behavioral Intention | 0.804 | 0.803 | 0.043 | 18.614 | 0 |
| Clinical Tools | 0.66 | 0.659 | 0.058 | 11.467 | 0 |
| Effort Expectancy | 0.772 | 0.767 | 0.051 | 15.171 | 0 |
| Performance Expectancy | 0.665 | 0.665 | 0.05 | 13.394 | 0 |

# Table A.8 Partial Least Squares Construct Reliability and Validity Report generated using SmartPLS v. 3.2.9 PLS

|  | Cronbach’s Alpha | rho_A | Composite Reliability | Average Variance Extracted (AVE) |
| --- | --- | --- | --- | --- |
| Anxiety | 0.617 | 0.768 | 0.786 | 0.568 |
| Attitude Toward Using Technology | 0.752 | 0.755 | 0.89 | 0.801 |
| Behavioral Intention | 0.756 | 0.761 | 0.891 | 0.804 |
| Clinical Tools | 0.489 | 0.501 | 0.795 | 0.66 |
| Effort Expectancy | 0.713 | 0.777 | 0.871 | 0.772 |
| Performance Expectancy | 0.755 | 0.784 | 0.856 | 0.665 |

# Figure B.1 Semi-structured Interview Guide


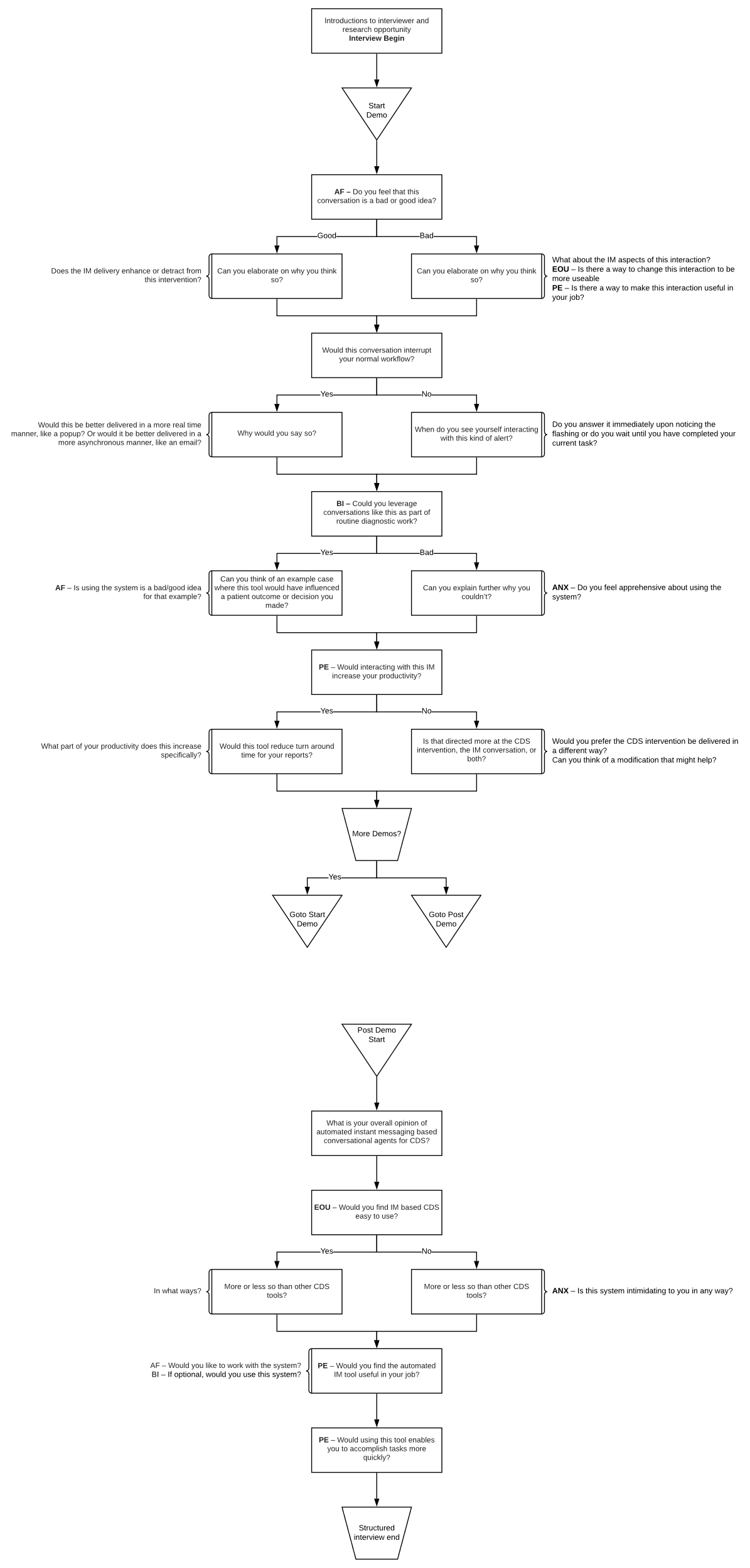


# Table B.2 Qualitative Analysis Labels

| Label | Further details |
| --- | --- |
| Expected effort increases | The amount of effort or work increases. This task would make my job more difficult. My interactions with the system would not be clear and understandable. *time increasing* |
| Expected effort the same |  |
| Expected effort decreases | The amount of effort decreases. This would make my job easier. My interactions with the system would be clear and understandable. *Time decreasing* |
| Positive attitude | I like this, this makes me happy |
| Neutral attitude |  |
| Negative attitude | I hate this, I do not like this |
| Positive intent to use | I would use this. I can see how this would be useful. This fits into my workflow. |
| Negative intent to use | I couldn’t use this. This would hurt my workflow. |
| Improve patient outcomes |  |
| No change in patient outcomes |  |
| Decrease patient outcomes |  |
| Increases anxiety | Increasing worry, unease, nervousness |
| Decreases anxiety | Decreasing worry, unease, nervousness |
| Expected performance increases | This would make my work faster. This would improve patient outcomes. Quality of work would be better. |
| Expected performance the same |  |
| Expected performance decreases | This would make my work slower. Quality of work would be worse. |
| Radiologist training | Student, trainee, resident, fellow |
| Seasoned radiologist | Old, been a radiologist for a long time |
| New radiologist | Young, new radiologist or new to the group |
| Trainee | Resident/fellow |
| Chat correct intervention | I like the conversational interface. I think a chatbot makes sense. |
| Chat incorrect intervention | I would prefer this intervention in a different format. |
| Example use given | Radiologist gave an example of how they would use the tool |

# Table B.3 Participant and Krippendorf's CU Alpha. Highlights show remaining narratives after dropping for poor inter-rater agreement, generated using Atlas.ti.

| Participant |  | K Cu Alpha |
| --- | --- | --- |
| 1 |  | 0.487 |
| 2 |  | 0.823 |
| 3 |  | 0.09 |
| 4 |  | 0.542 |
| 5 |  | 0.611 |
| 6 |  | 0.784 |
| 7 |  | 0.596 |
| 8 |  | 0.87 |
| 9 |  | 0.937 |
| 10 |  | 0.66 |
| 11 |  | 0.966 |
| 12 |  | 0.752 |
| 13 |  | 0.953 |
| 14 |  | 0.957 |
| 15 |  | 0.677 |
| 16 |  | 0.706 |
| 17 |  | 0.787 |
| 18 |  | 0.886 |
| 19 |  | 0.666 |
| 20 |  | 0.837 |
| 21 |  | 0.646 |
| 22 |  | 0.49 |
| 23 |  | 0.757 |

# B.4 Code Co-occurrence tables and Sankey Diagrams by Hypothesis

Code co-occurrence tables and Sankey diagrams generated using Atlas.ti. Gr is the groundedness of code, equal to the total number of quotes linked to a code.

## Hypothesis 1

*Expected effort is not a contributing factor in attitude towards the intervention but is a contributing factor in intent to use the intervention.
Null: Expected effort is a contributing factor in attitude towards the intervention and intent to use the intervention.*

Interactions between effort and attitude represent 16.01% of all attitude contributors. Interactions between effort and intent to use represent 15.93% of all intent to use contributors. Looking at the second level interactions, there are significant paths between effort, attitude, and intent to use. We are unable to reject the null hypothesis.

*Table B.4.1 Effort and Attitude/Intent to Use interactions*

|  | **Expected Effort Decreases** **Gr=272** | **Expected Effort Increases** **Gr=100** | **Expected Effort the Same** **Gr=34** |
| --- | --- | --- | --- |
| **Negative Attitude Gr=128** | 0 | 12 | 0 |
| **Neutral Attitude Gr=81** | 3 | 3 | 4 |
| **Positive Attitude Gr=622** | 103 | 20 | 0 |
| **Negative Intent to Use** **Gr=45** | 0 | 1 | 0 |
| **Positive Intent to Use** **Gr=363** | 60 | 11 | 0 |

*Figure B.4.1 First level interactions Sankey diagram Effort and Attitude/Intent to Use interactions*


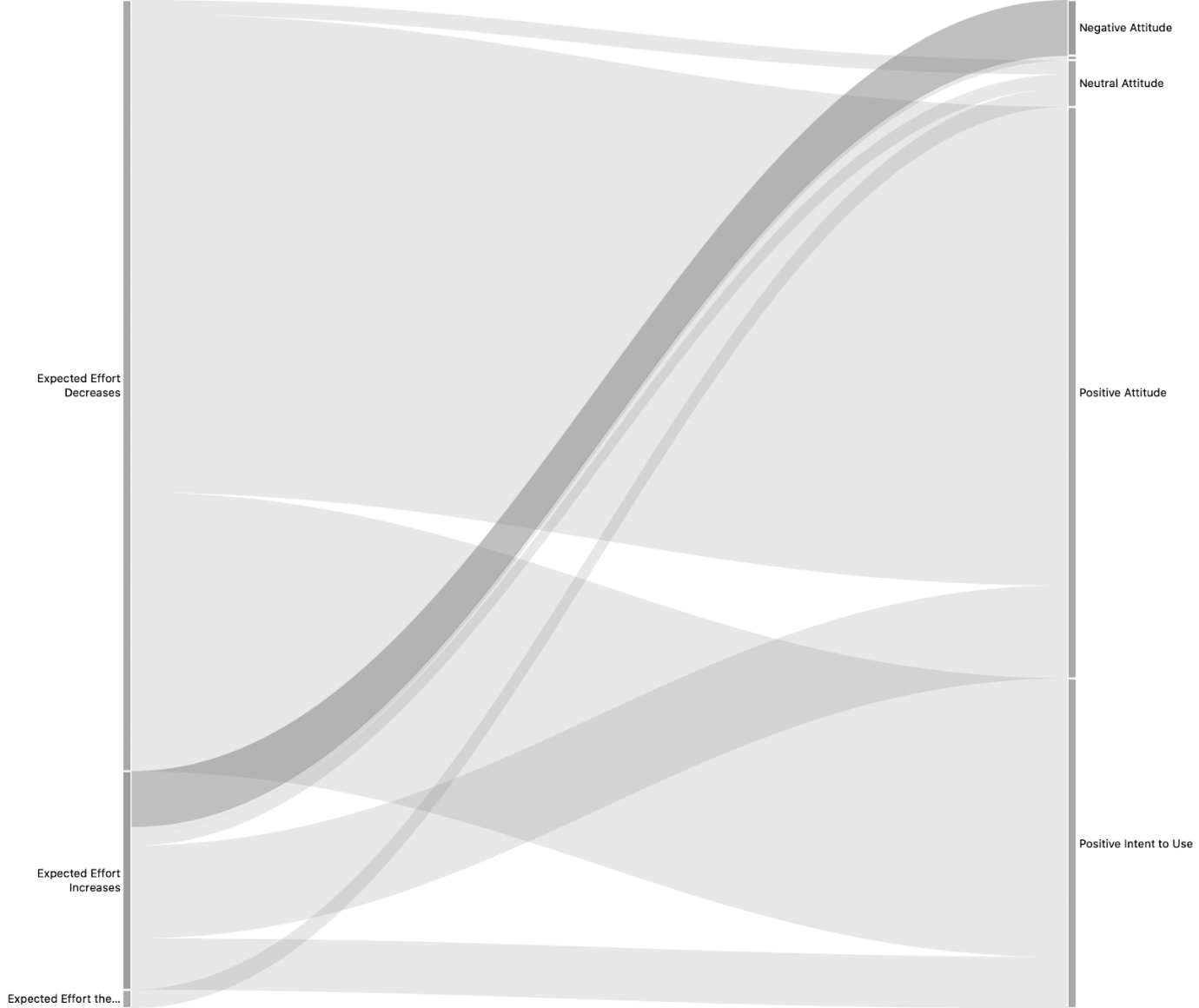


*Figure B.4.2 Second level interactions Sankey diagram Effort and Attitude/Intent to Use interactions*


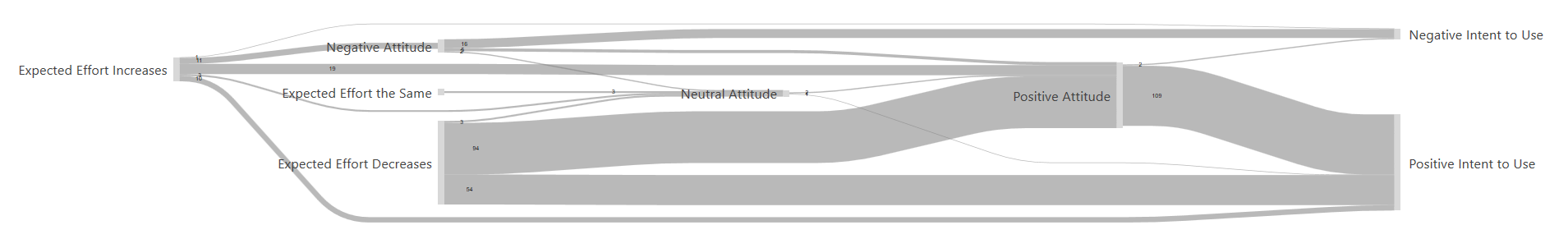


## Hypothesis 2

*With respect to this intervention, anxiety has a negative relationship with performance expectancy. As anxiety increases, performance expectancy decreases. As anxiety decreases, performance expectancy increases.
Null: Anxiety has no relationship or a positive relationship with performance expectancy. As anxiety increases, performance expectancy stays the same/increases. As anxiety decreases, performance expectancy stays the same/decreases.*

Overall anxiety is not commonly represented well within the interviews. There is not enough evidence to reject the null hypothesis.

*Table B.4.2 Anxiety and Performance interactions*

|  | **Decreases Anxiety Gr=54** | **Increases Anxiety Gr=43** |
| --- | --- | --- |
| **Expected Performance Decreases Gr=55** | 0 | 0 |
| **Expected Performance Increases Gr=381** | 4 | 2 |
| **Expected Performance the Same Gr=43** | 0 | 0 |

*Figure B.4.3 First level interactions Sankey diagram Anxiety and Performance interactions*


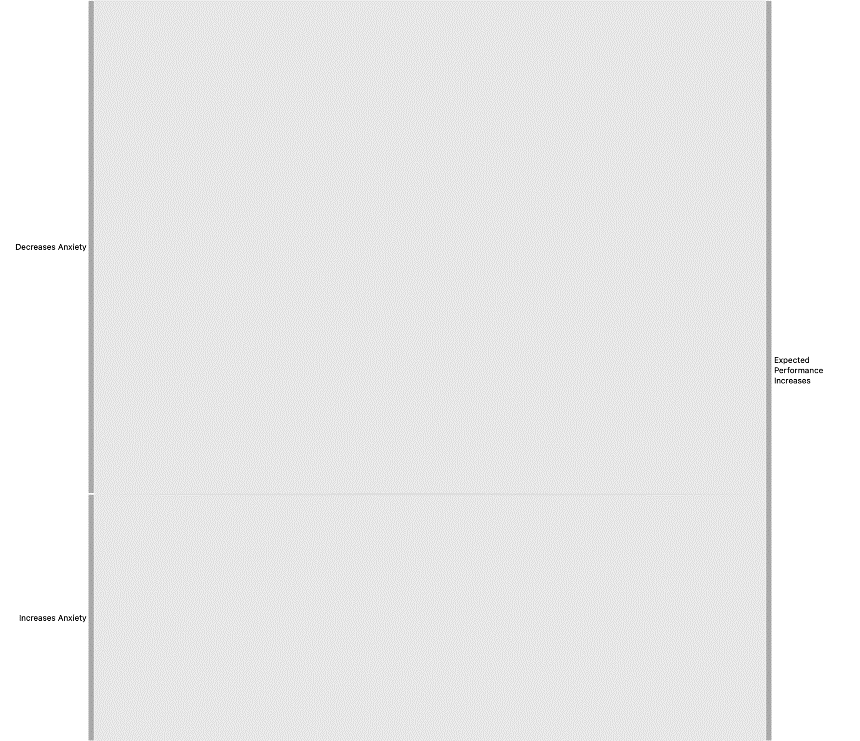


## Hypothesis 3

*Radiologists have a positive attitude towards this intervention and a high intent to use this intervention if it were produced.
Null: Radiologists have a negative attitude and/or a low intent to use this intervention if it were produced.*

Positive attitude is present 4.859 times more than negative (GR 622/128). Positive intent to use is present 8.067 times more than negative (GR 363/45). We can reject the null hypothesis.

*Table B.4.3 Attitude and Intent to Use interactions*

|  | **Negative Attitude Gr=128** | **Positive Attitude Gr=622** |
| --- | --- | --- |
| **Negative Intent to Use Gr=45** | 17 | 2 |
| **Positive Intent to Use Gr=363** | 0 | 134 |

*Figure B.4.4 First level interactions Sankey diagram Attitude and Intent to Use*


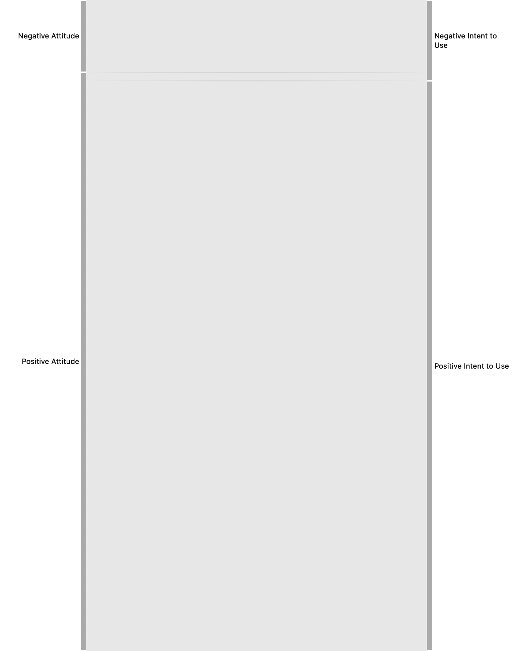


## Hypothesis 4

For this intervention, intent to use and attitude are mostly influenced by performance expectancies.

### Hypothesis 4a

*Radiologist’s attitude towards this intervention is mostly influenced by the expected performance of the system.
Null: Radiologist’s attitude towards this intervention is mostly influenced by the other factors of the system.*

Attitude correlates mostly with effort (17.45%), followed by performance (15.40%), and then anxiety (4.69%). We are not able to reject the null hypothesis.

*Table B.4.4 Attitude and Anxiety/Expected Effort/Expected Performance interactions*

|  | **Negative Attitude Gr=128** | **Neutral Attitude Gr=81** | **Positive Attitude Gr=622** |
| --- | --- | --- | --- |
| **Increases Anxiety Gr=43** | 9 | 2 | 12 |
| **Decreases Anxiety Gr=54** | 0 | 0 | 16 |
| **Expected Effort Decreases Gr=272** | 0 | 3 | 103 |
| **Expected Effort Increases Gr=100** | 12 | 3 | 20 |
| **Expected Effort the Same Gr=34** | 0 | 4 | 0 |
| **Expected Performance Decreases Gr=55** | 5 | 7 | 23 |
| **Expected Performance Increases Gr=381** | 0 | 5 | 84 |
| **Expected Performance the Same Gr=43** | 0 | 0 | 4 |

*Figure B.4.5 First level interactions Sankey diagram Attitude and Anxiety/Expected Effort/Expected Performance interactions*


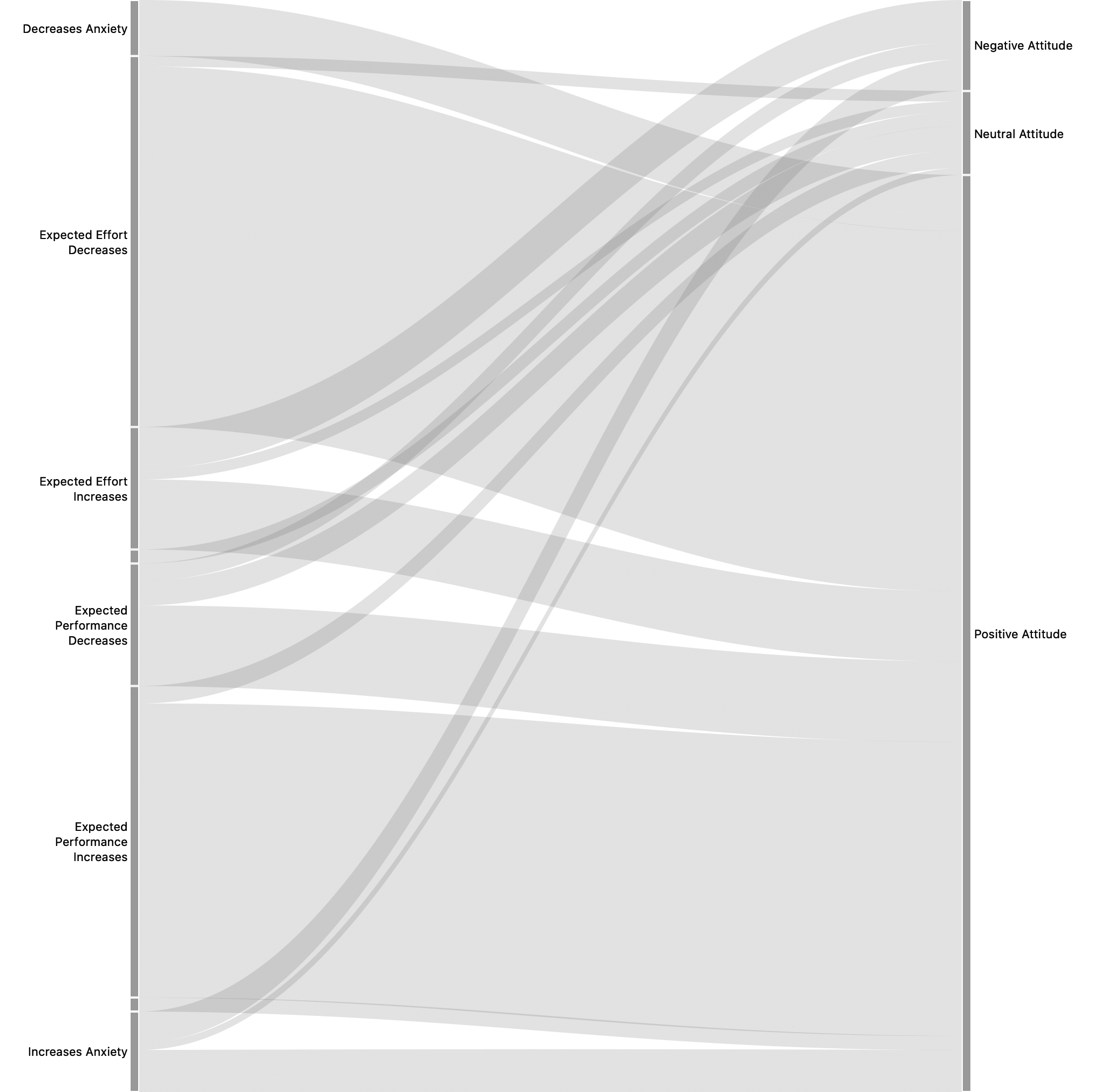


## Hypothesis 4b

*Radiologist’s intent to use this intervention is mostly influenced by expected performance of the system.
Null: Radiologist’s intent to use this intervention is mostly influenced by other factors of the system.*

Intent to use correlates mostly with attitude (37.75%), performance (18.38%), expected effort (17.65%), and anxiety (2.94%). We are not able to reject the null hypothesis.

*Table B.4.5 Intent to Use and Anxiety/Expected Effort/Expected Performance/Attitude interactions*

|  | **Negative Intent to Use Gr=45** | **Positive Intent to Use Gr=363** |
| --- | --- | --- |
| **Decreases Anxiety Gr=54** | 0 | 8 |
| **Increases Anxiety Gr=43** | 4 | 0 |
| **Expected Effort Decreases Gr=272** | 0 | 60 |
| **Expected Effort Increases Gr=100** | 1 | 11 |
| **Expected Effort the Same Gr=34** | 0 | 0 |
| **Expected Performance Decreases Gr=55** | 6 | 2 |
| **Expected Performance Increases Gr=381** | 0 | 62 |
| **Expected Performance the Same Gr=43** | 3 | 2 |
| **Negative Attitude Gr=128** | 17 | 0 |
| **Neutral Attitude Gr=81** | 0 | 1 |
| **Positive Attitude Gr=622** | 2 | 134 |

*Figure B.4.6 First level interactions Sankey diagram Intent to Use and Anxiety/Expected Effort/Expected Performance/Attitude interactions*


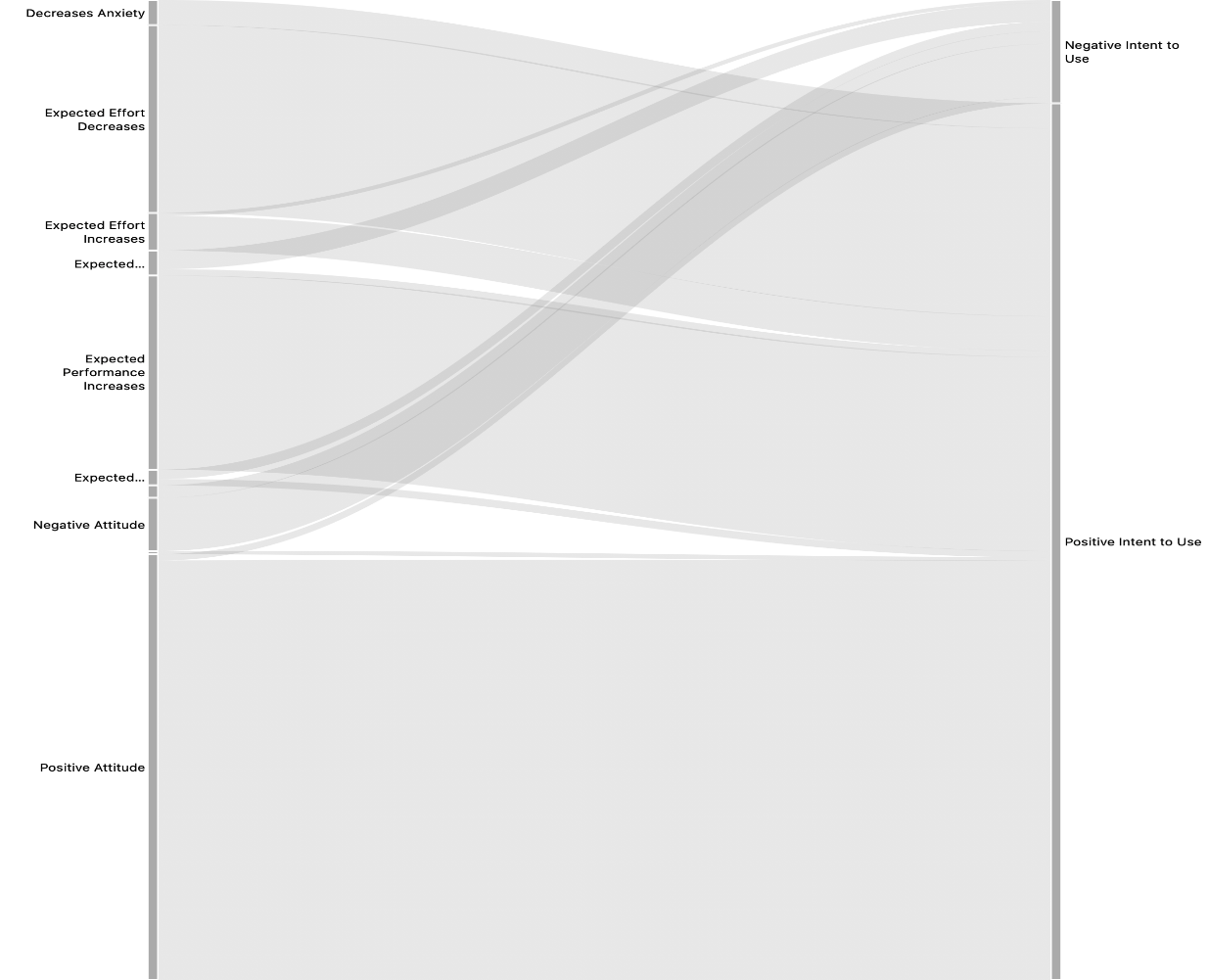


## Hypothesis 5

*Radiologist’s performance expectancies positively influence their effort expectancies. As expected performance increases, expected effort decreases.
Null: Radiologist’s performance expectancies don’t influence or negatively influence their expectancies surrounding effort. As performance increases, expected effort stays the same or increases.*

Overall, performance is correlated with effort in 41.87% of quotes. Performance increases to effort decreasing 44.12%, effort increasing 13%, and effort the same 32.35%. Performance decreases to effort increasing 20% and decreases/the same 0%. We can reject the null hypothesis.

*Table B.4.6 Expected Performance and Expected Effort interactions*

|  | **Expected Performance Decreases** **Gr=55** | **Expected Performance Increases** **Gr=381** | **Expected Performance the Same** **Gr=43** |
| --- | --- | --- | --- |
| **Expected Effort Decreases** **Gr=272** | 0 | 120 | 4 |
| **Expected Effort Increases** **Gr=100** | 20 | 13 | 2 |
| **Expected Effort the Same** **Gr=34** | 0 | 11 | 0 |

*Figure B.4.7 First level interactions Sankey diagram Expected Performance and Expected Effort interactions*


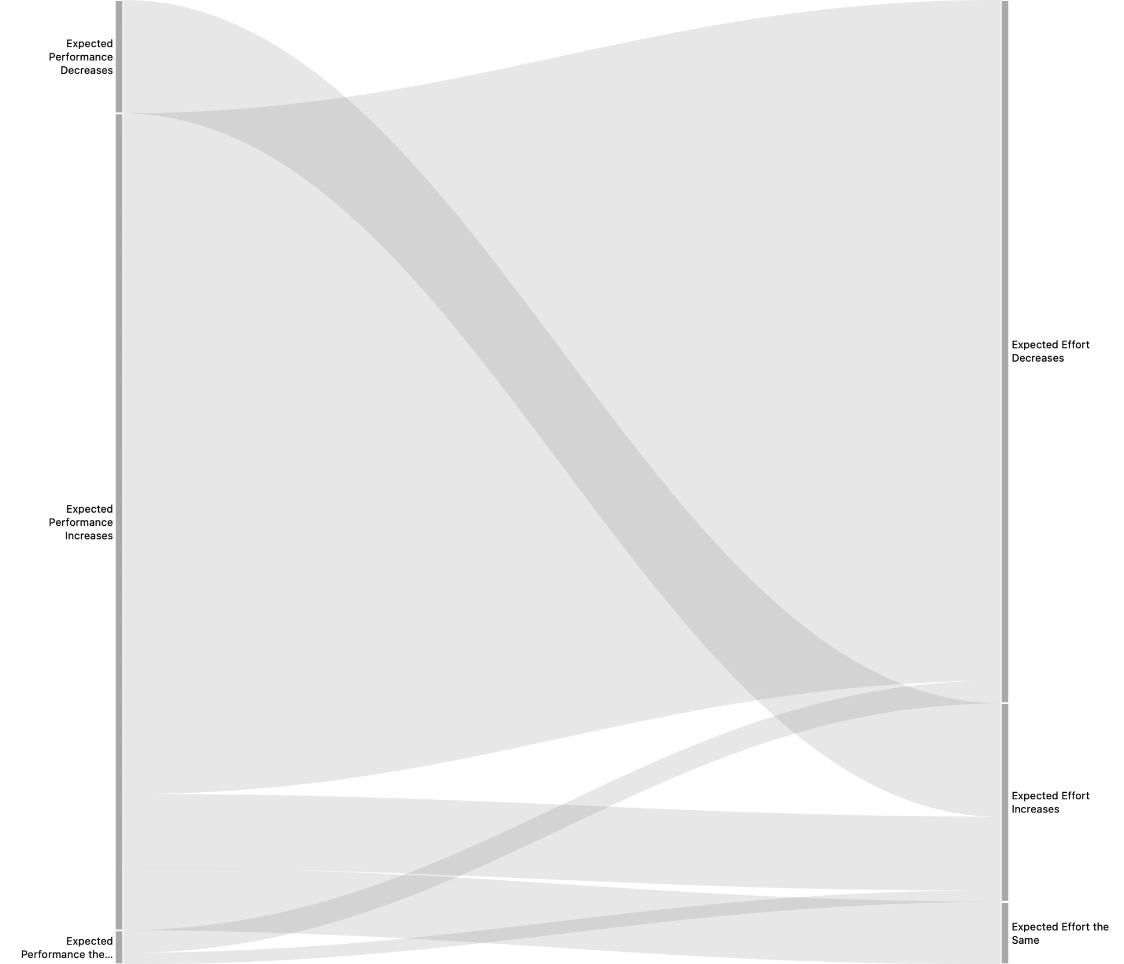


# B.5 Additional quotes representing common themes

## Expected effort influence on attitude

- Reducing time to acquire and apply clinical knowledge.
- “…however many seconds it takes for everyone…and it's different for everyone to figure out how they want to go about finding this information. We all kind of I think most people know where to look IE the ACR guidelines, but…having this thought process kind of forcing us to focus on this dialog box kind of streamlines that whole process. So I think overall, it should enhance the workflow”
- “…these are the things when we're not given enough information…Some people perseverate on the lack of information more than others. And some people are really dutiful and want to go into the EMR and look, but that could be one to two minutes, and then compound that over an entire shift. Do that a couple times. That could be an hour that you've saved if you had this information in a ready format, or in a readily available format, so I think this definitely makes you, from this particular type of interactions definitely makes you more efficient, I believe.”
- Increasing multitasking
- “I think it is a great idea. I think it helps you do multiple things, maybe not just in this cardiac workup. But like for lung nodules when your kind of trying to decide what the appropriate workup is. We always have a caveat that takes seconds to say but it's still seconds that you have to say it every time. You know, if the patient has high risk for pulmonary malignancy recommend whatever. We know that they're already high risk for whatever, then I feel like we don't need to say that. Or if that even auto populates the patient has these risk factors that we would recommend discussing these risk factors.”
- Trusting CDSS as safety nets
- “…we touted on AI is not to replace your diagnostic skills, but eight other things, whether it's making you more efficient or providing kind of a little safety net, right. Maybe you forgot to mention a follow up or something that really should be a critical result.”

## Expected performance as the major influencer of attitude and intent to use

- Radiologists expect to be interrupted or context switch quickly.
- “Interrupt My normal workflow? Well, I guess it depends on what is normal. This would not interrupt my normal workflow. We’re constantly getting interrupted. It would just be another interruption among a series of normal interruptions.”
- “…this is kind of the thought process that I, this is I go through this checklist. Basically, every time I close a study, we look at the work list again. I’m thinking to myself, looking over my shoulder at the residents and looking at their, their work list, and thinking about [county hospital] over here, looking at how deep my work list is how far so I basically run this checklist mentally, in between each exam.”
- Reducing effort is highly embraced.
- “I love the idea that I’m not having to call someone and that automatically reminds me and I can just either do one click and go one click would be nice to just be like, Yes.”
- “the status quo is quicker or definitely is quicker than then that interface I just saw on the video.”
- “…I’m responsible for all those things that I don’t see on a regular basis…Let me go back to that algorithm figure out what I need to say. This would be a really great tool for me in those cases, because I don’t have to worry as much I think I’m missing a recommendation or something like that. I don’t have to hedge as much. I don’t have to hurry try to get to get to my list. Luckily, we don’t aren’t too inundated so it’s not an issue but I do feel like this will help me to put the appropriate things in with the appropriate recommended”
- “Well, it would shortcut having to call a technologist and initiate a conversation about what the patient was, what the study was, the post processing that you needed, done. So if the radbot could predict that you might need it. And could figure out what you needed quicker and would negate having that phone call that would be positive.”
- Radiologists will trade effort for performance.
- “So it slows you down slightly, but in the long run of collections and all that stuff. Yeah, I think it would [improve performance], because you’re making sure you get reimbursed and get the correct RVU amounts for the right study.”
- “… the amount of time it takes to look those things up, and it’s not super frequent, but not super infrequent…either it’s taking you more time to figure it out or you also end up with more a variation amongst different radiologists for the recommendation. So you’re not only might save time, but you might also decrease the heterogeneity of the recommendations. And probably more, you’d be probably more likely to actually be following the guidelines since you’d be prompted to to adhere to them.”
- “…I think maybe it slows you slightly on the front end, but on the back end, it helps you, and it helps clinicians too”
- “I think it would decrease my productivity very minimally. But for a good cause.”

## Overall

- “No, I think again, this is it's all these videos have demonstrated processes which are mostly done mentally by all radiologists. And again, it's not always easy to kind of put these things on a screen or you know, because your kind of you're, you're juggling a couple different priorities at the same time. So, I think this is kind of taking an existing thing and it's making a more organized and streamlined fashion.”
- “Yeah, assuming that you would have gone to the EMR it was important enough to go there. And if you went there to take more time, okay. But at the same point, you know, it might change the threshold at which you would ask a question, right? It's like, it'd be nice if we knew this and it's easy just to query it. But you otherwise might not go to the EMR.”
- “I guess the part that causes me to pause is who's going to be mining for new updates? And how can we be sure that we're staying current on recommendations? You know, how is that? Who's going to handle that part of it?”

# C.1 Extended Background - Standards in Radiology CDSS

“In most health care settings, radiology departments were the groundbreakers and leaders in clinical information technology (IT), and at many sites, radiologists and imaging informaticists were responsible for specifications, purchase, implementation, and sometimes even creation of these technologies, while also carrying the ongoing burdens of training, maintenance, support, and operations for these systems.” Kohli, Dreyer, and Geis describe the evolution of informatics in radiology, with the advent of digital modalities in the 1970s and the migration into the fully digital realm of PACS, RIS, Voice Recognition (VR), EMR, and CDSS [41]. This evolution led to the generation of standards that enable CDSS. An understanding of the standards outlined below is useful in developing decision support software for the radiology workflow.

### Radiology Workflow Statuses

Each status described in table C.1.1 is a unique time point for a radiology exam. Patients can have many different statuses, each unique to the exam being processed. Each status has relevant facts that can be accessed for CDSS tools.

| Status | Notes | Sending Systems | Facts |
| --- | --- | --- | --- |
| Ordered | The ordering physician has input exam details into the CPOE system. | CPOE in EMR or RIS | - Ordering physician name, National Provider Identifier (NPI) - Order entry timestamp - Exam schedule timestamp - Patient name, Medical Record Number (MRN) - Patient location - Modality - Procedure code and description - Exam accession number |
| Image acquisition Start | The technologist has begun the exam and is acquiring medical images of the patient. | Modality, RIS, or PACS | - Technologist name and identifier - Exam begin timestamp - Modality Application Entity Title (AET) - Exam accession number |
| Image acquisition completed | The technologist has completed the exam of the patient. | Modality, RIS, or PACS | - Technologist name and identifier - Technologist comments - Exam ended timestamp - Modality Application Entity Title (AET) - Exam accession number |
| Dictation start | Attendee or trainee has begun reviewing the exam and is dictating a report. | VR | - Attending radiologist name, NPI - Trainee name, NPI - Dictation begin timestamp - Exam accession number |
| Dictation complete | Attendee or trainee has finished dictating the report and submitted a draft report. | VR | - Attending radiologist name, NPI - Trainee name, NPI - Dictation end timestamp - Exam accession number - Draft report text |
| Finalized | Attending radiologist has final signed the report and committed to the EMR. | VR | - Attending radiologist name, NPI - Trainee name, NPI - Final sign timestamp - Exam accession number - Report text |
| Addendum | Attending radiologist has entered additional information supplementing the finalized report. | VR | - Attending radiologist name, NPI - Trainee name, NPI - Addendum timestamp - Exam accession number - Addendum text |
| Canceled | Exam was cancelled by the ordering physician, technologist, radiologist, or other staff for various reasons. | EMR, RIS, or PACS | - Exam accession number |

Table C.1.1 Study Statuses, Sending Systems, and Facts

### Health Level Seven Version 2

“HL7’s Version 2.x (V2) messaging standard is the workhorse of electronic data exchange in the clinical domain and arguably the most widely implemented standard for healthcare in the world. This messaging standard allows the exchange of clinical data between systems. It is designed to support a central patient care system as well as a more distributed environment where data resides in departmental systems [83].” HL7v2 is a dominant communications standard for EMR systems. HL7v2 messages are triggered by clinical events, including all status messages described above. Full descriptions of the HL7v2 standard, how to read and interpret HL7, and how it is utilized in clinical imaging are described on the cited HL7.org reference site.

### Digital Imaging and Communications in Medicine

DICOM standards describe metadata surrounding images. This is inclusive of all image acquisition timestamps, modality specific configurations, and image information [84]. PACS stores DICOM formatted images, and external applications can query and retrieve images and associated metadata through PACS. Web Access to DICOM Objects (WADO) can enable web applications to display DICOM objects from the PACS with images and metadata [85].

### Application Programming Interfaces

Application Programming Interfaces (API) are defined protocols to interface with a system [86]. DICOM for example has defined API surrounding image query/retrieve functions. Representational State Transfer (REST) is an API utilized in web services development. RESTful interfaces have 5 main functions – GET, POST, PUT, PATCH, and DELETE – to build programmatically interactive web interfaces [87]. Health Level 7’s Fast Health Interoperability Resources (FHIR) is an API interface standard for accessing EMR data that includes a RESTful web interface [88]. FHIR’s integration with radiology systems is growing [89, 90], and the availability of EMR data through FHIR interfaces are providing new avenues for development of CDSS.

### Workflow Engines

Workflow Engines (WFE) are tools that enable workflows by interpreting events and applying logic to generate new events, and are common tools to manage workflow outside of healthcare [8]. In their description of a DICOM-enabled WFE, Erickson, Langer, Blezek, Ryan, and French describe the generic WFE as “…a workflow engine is a technology designed to coordinate humans and automation to accomplish a task. Workflow engines typically have a database within them, but they also classify work items with one or more metatags. Such tags are then used by an “orchestrator” process to direct the work item to one or more work steps, enabling more flexible work patterns, user-friendly descriptions, and superior methods for error recovery. Flexibility is achieved since new tags and algorithms can be created without changing the database schema.” Through the use of DICOM metadata, Erickson et al. accomplished 98% compliance rate of automated brain tumor comparison prior to radiologist review, compared to 29% prior to implementation [91]. This automation of CAD analysis is a clear enhancement to workflow, providing radiologist with pertinent information in a timely fashion to improve clinical care. However, it is limited to DICOM workflow and PACS specific integration; and cannot create workflow events outside of this paradigm.

*Implementation of Workflow Engine Technology to Deliver Basic Clinical Decision Support Functionality* investigates implementing an open source WFE and workflow editor to support CDSS in the EMR. Huser, Rasmussen, Oberg, and Starren’s implementation is valuable for understanding both the underlying technical foundation of WFE as well as the non-programmer operator components of the system. The salient arguments are as follows.

- Workflow logic should be presented in a digestible format (i.e. flow charts) that non-programmers can use to build, review, and understand workflow interventions. Logic falls into 3 categories with varying level of expertise required to build interventions in any category.
  - Simple – few categorical input and output parameters, resolving basic interactions
  - Advanced – increase in complexity, increase in number and diversity of categorical and continuous parameters. Concept of time bounded/temporal event logic is introduced in this level.
  - Ultimate – additional complexity increase, numerical and logical operations on parameter sets. Inclusion of healthcare ontologies [such as Radlex [92] radiology specific ontology inclusive of workflow event terms] and terminology extraction based parameters (Common Data Elements for Radiology [93] provides radiology centric parameters). Complex, looping logic can be utilized by expert level WFE users.
- Ability to evaluate CDSS implementations in the WFE using retrospective data, implying that it is necessary for WFE to be connected to data warehouses. Evaluation should include:
  - Percentage of testing cohort with a generated event
  - How does the CDSS intervention perform when tested against additional cohorts
  - Should the inclusion/exclusion criteria be modified based on the results of the testing
- CDSS interventions should be tested in a prospective function prior to deployment. This can be modeled with retrospective data as necessary.
- WFE should be interoperable with other logic systems
- WFE must be built to operate on cross-industry workflow standards [such as the radiology specific Open Computer-Assisted Radiologist Decision Support (OpenCARDS) resource [20]]

These are among many other recommendations that should serve as evaluative criteria for any WFE in healthcare [94].

#### At Time of Radiology Exam Ordering

Radiology exam ordering can be misunderstood by clinicians. Errors in ordering include inappropriate orders - whether that means scanning the wrong anatomy, scanning with the wrong technique, or harmful exposure to contrast materials - which can lead to over or under utilization of imaging exams [95]. Computerized physician order entry (CPOE) systems are designed not to replace physician’s knowledge of imaging but to guide clinicians as they select exams that are clinically appropriate and are billable [96]. By providing information on costs and risks, CPOE can influence clinical workflows [97]. CPOE systems have promising results in reducing redundant orders and overall imaging utilization in support of standards driven patient care [98, 99]. By integrating clinical history and indications into imaging exam ordering, CPOE enhances radiologist-clinician communications forcing relevant clinical details to be included at time of ordering [100].

Radiologists want to participate CPOE development to increase appropriate imaging, decrease inappropriate imaging, answer clinician questions without human intervention, and to bring relevant health record data into the imaging workflow [101]. Chan et al. describe the radiologists’ involvement in CPOE development in building guidelines to support CPOE interfaces, “The backbone of the clinical decision support software is the appropriate use criteria…Appropriate use criteria are evidence-based clinical practice guidelines that assist professionals who order and furnish advanced diagnostic imaging services…” The authors advocate the use of the American College of Radiology Appropriateness Criteria for developing guidelines that can be implemented by many vendors [102, 103].

#### After Image Acquisition

Once the images have been acquired, there is a variety of data that the radiologist must combine and interpret. CDSS and AI tools can be augmenting the data that is presented to the radiologist. Examples of such augmentation are found in Computer-Aided Detection (CAD), radiomics, and clinical history analysis.

CAD/Computer-Aided Analysis and Computer-Aided Diagnosis (CADx) are paradigms that have been described since 1959 [7] and implemented in various ways since. Agarwal, Erickson, and Kahn describe CAD as a system to reduce radiologist errors while increasing accuracy and efficiency. CAD’s history mimics the history of computer science, with early implementations designed with static code and modern solutions completed with machine learning and AI. Regardless of technology used to implement, CAD systems “perform four critical steps: preprocessing, segmentation, region of interest (ROI) analysis, and determination…” [8] CAD systems can exist outside of the PACS environment, requiring manual intervention by a radiologist or other staff to process [9]. CAD systems are not inherently CDSS, however, they can be incorporated into CDSS [10]. Highly integrated CAD systems can affect workflow by directing attention to automatically generated findings through PACS presentation layer modifications [11] or displaying algorithmically generated scoring metadata [12].

Radiomic systems differ from CAD, in that they are not trying to answer a clinical question but are trying to gather as much quantitative data as possible [104]. “Radiomics – the high-throughput computation of quantitative image features extracted from medical imaging modalities- can be used to aid clinical decision support systems in order to build diagnostic, prognostic, and predictive models, which could ultimately improve personalized management based on individual characteristics [105].” Quantitative features include shape/geometric, first-order statistics voxel intensities, second-order statistics textural, and higher-order statistics derived from applied filters [106].

Presenting prior clinical history at time of radiology read is diagnostically valuable [13-16]. In their pursuit of creating a history reporting tool, Gorniak et al. describe the time consuming workflow a radiologist must pursue to acquire a complete patient history, through the use of “patient questionnaires, prior radiology reports, hospital information systems, EMR, calls to the referring provider, and patient interviews.” Their tool uses Natural Language Processing (NLP) algorithms to retrieve relevant clinical information from prior radiology studies and present this as part of the PACS interface [17]. While clinical history is valuable, the current methods to collect and interpret non-PACS history data is not integrated into the radiology workflow.

#### Intra-report Analysis

Boland et al. describe a system, like CPOE, that uses evidence-based guidelines to embed decision support into the radiology report generation workflow. The authors describe a system that would require the radiologist to step through decision support trees when building reports. Such a system would enhance clinical outcomes and reduce implementation time of new evidence supported guidelines [18]. *In Automatic Retrieval of Bone Fracture Knowledge Using Natural Language Processing*, the authors built a system to interpret radiologist reporting in real time and provide automated feedback. The authors implemented this in a separate, web based system outside of the radiology workflow [19].

#### Post-report Analysis

Once the radiologist has completed their report, the output is a blob of unstructured text. Interventions in post-report analysis include extracting categorical data, radiologist-clinician communication, and quality improvement systems.

Within the unstructured text can be a multitude of categorical data ripe for mining and output into CDSS. Evaluation criteria such as Response Evaluation Criteria in Solid Tumors (RECIST), enable decision support systems downstream of the radiology report. “…although many radiology reports contain the necessary [for RECIST] measurements, variability among reporting styles makes harvesting this information difficult and costly [to derive clinical facts] [20].” NLP tools have been created to extract generated clinical facts in these scoring systems and present them to clinicians to augment clinical workflows [21, 23]. Patel et al. describe a system using NLP techniques to merge radiology and pathology unstructured reports, enhancing clinical relevance by combining the context of these independently generated results [24].

In 2010’s *The Future Role of Radiology in Healthcare*, the European Society of Radiology advocates strongly that radiologists should network with clinicians, participate in discussions with patients and physicians, consult at multidisciplinary conferences, and use technology like video-chat to enhance tele-radiology [25]. Weiss, Kim, Branstetter, and Prevedello outline requirements for ‘closed-loop’ radiology results communication, describing the critical factors as timing, format, and system level audit trail including acknowledgement of receipt [26]. Synchronous and asynchronous communication systems have been developed for results notifications, including: AI/NLP based tools that automatically review unstructured report text and suggest communication opportunities, video chat, paging, faxing, email, and phone systems [27-30].

Quality improvement initiatives function through retrospective analysis of radiologist’s reports. AI and CDSS tools can be applied to help drive this analysis. Reiner describes an AI tool that intakes original and peer reviewed reports and outputs comparative analytics, creating objective feedback for the report initializer [31]. In a later article, Reiner describes using radiology reports to report on uncertainty and outlines the need for real-time feedback to the radiologist [32].

#### Radiology Workflow Adjacent

Workflow adjacent CDSS enhance the performance of the radiologist and range from activities static to the clinical workflow, such as structured report templates, decision trees, and ontologies [20], to real-time workflow prioritization, management, and feedback tools [33]. For the purposes of this review, static decision support technologies are not reviewed. Real-time or near real-time workflow CDSS tools generally function by acquiring clinical data by listening to Health Leven 7 (HL7) [39] or Digital Imaging and Communications in Medicine (DICOM) [40] interfaces, or through Extract, Transfer, Load (ETL) [107] techniques. These tools monitor imaging workflow statuses (ordered, image acquisition start, image acquisition completed, dictation start, dictation complete, finalized, addendum, and canceled), tracking timestamps for each [34]. Outputs in the form of dashboards can be integrated into PACS and RIS or exist as separate web-based interfaces. Such dashboards are utilized to support staffing, quality improvement projects, and radiologist EMR integrations with varying efficacy [35-38].

References

1. Sutton RT, Pincock D, Baumgart DC, Sadowski DC, Fedorak RN, Kroeker KI. An overview of clinical decision support systems: benefits, risks, and strategies for success. npj Digital Medicine. 2020;3(1):17.

2. Choy G, Khalilzadeh O, Michalski M, Do S, Samir AE, Pianykh OS, et al. Current Applications and Future Impact of Machine Learning in Radiology. Radiology. 2018;288(2):318-28.

3. Dreyer KJ, Geis JR. When Machines Think: Radiology's Next Frontier. Radiology. 2017;285(3):713-8.

4. Gichoya JW, Alarifi M, Bhaduri R, Tahir B, Purkayastha S, editors. Using cognitive fit theory to evaluate patient understanding of medical images. 2017 39th Annual International Conference of the IEEE Engineering in Medicine and Biology Society (EMBC); 2017 11-15 July 2017.

5. Gichoya JW, Nuthakki S, Maity PG, Purkayastha S. Phronesis of AI in radiology: Superhuman meets natural stupidity. arXiv preprint arXiv:180311244. 2018.

6. Venkatesh V, Morris M, Davis G, Davis F. User Acceptance of Information Technology: Toward a Unified View. MIS Quarterly. 2003;27:425-78.

7. Lodwick GS, Turner AH, Jr., Lusted LB, Templeton AW. Computer-aided analysis of radiographic images. Journal of chronic diseases. 1966;19(4):485-96.

8. Agrawal JP, Erickson BJ, Kahn CE, Jr. Imaging Informatics: 25 Years of Progress. Yearbook of medical informatics. 2016;Suppl 1:S23-31.

9. Nowinski WL, Qian G, Hanley DF. A CAD System for Hemorrhagic Stroke. The neuroradiology journal. 2014;27(4):409-16.

10. Stivaros SM, Gledson A, Nenadic G, Zeng XJ, Keane J, Jackson A. Decision support systems for clinical radiological practice -- towards the next generation. Br J Radiol. 2010;83(995):904-14.

11. Wang Y, Yan F, Lu X, Zheng G, Zhang X, Wang C, et al. IILS: Intelligent imaging layout system for automatic imaging report standardization and intra-interdisciplinary clinical workflow optimization. EBioMedicine. 2019;44:162-81.

12. Barinov L, Jairaj A, Becker M, Seymour S, Lee E, Schram A, et al. Impact of Data Presentation on Physician Performance Utilizing Artificial Intelligence-Based Computer-Aided Diagnosis and Decision Support Systems. J Digit Imaging. 2019;32(3):408-16.

13. Berbaum KS, Franken EA, Jr. Commentary does clinical history affect perception? Acad Radiol. 2006;13(3):402-3.

14. Berbaum KS, Franken EA, Jr., Dorfman DD, Lueben KR. Influence of clinical history on perception of abnormalities in pediatric radiographs. Acad Radiol. 1994;1(3):217-23.

15. Leslie A, Jones AJ, Goddard PR. The influence of clinical information on the reporting of CT by radiologists. Br J Radiol. 2000;73(874):1052-5.

16. Reiner BI. Medical Imaging Data Reconciliation, Part 3: Reconciliation of Historical and Current Radiology Report Data. Journal of the American College of Radiology. 2011;8(11):768-71.

17. Gorniak RJ, Sevenster M, Flanders AE, Deshmukh SP, Ford RW, Katzman GL, et al. A PACS-Integrated Tool to Automatically Extract Patient History From Prior Radiology Reports. J Am Coll Radiol. 2016;13(10):1249-52.

18. Boland GW, Thrall JH, Gazelle GS, Samir A, Rosenthal DI, Dreyer KJ, Alkasab TK. Decision support for radiologist report recommendations. J Am Coll Radiol. 2011;8(12):819-23.

19. Do BH, Wu AS, Maley J, Biswal S. Automatic retrieval of bone fracture knowledge using natural language processing. J Digit Imaging. 2013;26(4):709-13.

20. Kohli M, Alkasab T, Wang K, Heilbrun ME, Flanders AE, Dreyer K, Kahn CE, Jr. Bending the Artificial Intelligence Curve for Radiology: Informatics Tools From ACR and RSNA. J Am Coll Radiol. 2019.

21. Liu Y, Zhu LN, Liu Q, Han C, Zhang XD, Wang XY. Automatic extraction of imaging observation and assessment categories from breast magnetic resonance imaging reports with natural language processing. Chin Med J (Engl). 2019;132(14):1673-80.

22. Esmaeili M, Ayyoubzadeh SM, Ahmadinejad N, Ghazisaeedi M, Nahvijou A, Maghooli K. A decision support system for mammography reports interpretation. Health Inf Sci Syst. 2020;8(1):17.

23. Bozkurt S, Gimenez F, Burnside ES, Gulkesen KH, Rubin DL. Using automatically extracted information from mammography reports for decision-support. Journal of biomedical informatics. 2016;62:224-31.

24. Patel TA, Puppala M, Ogunti RO, Ensor JE, He T, Shewale JB, et al. Correlating mammographic and pathologic findings in clinical decision support using natural language processing and data mining methods. Cancer. 2017;123(1):114-21.

25. European Society of R. The future role of radiology in healthcare. Insights Imaging. 2010;1(1):2-11.

26. Weiss DL, Kim W, Branstetter BFt, Prevedello LM. Radiology reporting: a closed-loop cycle from order entry to results communication. J Am Coll Radiol. 2014;11(12 Pt B):1226-37.

27. Larson PA, Berland LL, Griffith B, Kahn CE, Jr., Liebscher LA. Actionable findings and the role of IT support: report of the ACR Actionable Reporting Work Group. J Am Coll Radiol. 2014;11(6):552-8.

28. Meng X, Ganoe CH, Sieberg RT, Cheung YY, Hassanpour S. Assisting radiologists with reporting urgent findings to referring physicians: A machine learning approach to identify cases for prompt communication. Journal of biomedical informatics. 2019;93:103169.

29. Lacson R, Prevedello LM, Andriole KP, O'Connor SD, Roy C, Gandhi T, et al. Four-year impact of an alert notification system on closed-loop communication of critical test results. AJR Am J Roentgenol. 2014;203(5):933-8.

30. Rosenkrantz AB, Sherwin J, Prithiani CP, Ostrow D, Recht MP. Technology-Assisted Virtual Consultation for Medical Imaging. J Am Coll Radiol. 2016;13(8):995-1002.

31. Reiner BI. Redefining the Practice of Peer Review Through Intelligent Automation-Part 3: Automated Report Analysis and Data Reconciliation. J Digit Imaging. 2018;31(1):1-4.

32. Reiner BI. Quantifying Analysis of Uncertainty in Medical Reporting: Creation of User and Context-Specific Uncertainty Profiles. J Digit Imaging. 2018;31(4):379-82.

33. Burns JL, Hasting D, Gichoya JW, McKibben B, 3rd, Shea L, Frank M. Just in Time Radiology Decision Support Using Real-time Data Feeds. J Digit Imaging. 2019.

34. Chen R, Mongkolwat P, Channin DS. RadMonitor: radiology operations data mining in real time. J Digit Imaging. 2008;21(3):257-68.

35. Nance JW, Jr., Meenan C, Nagy PG. The future of the radiology information system. AJR Am J Roentgenol. 2013;200(5):1064-70.

36. Nagy PG, Warnock MJ, Daly M, Toland C, Meenan CD, Mezrich RS. Informatics in radiology: automated Web-based graphical dashboard for radiology operational business intelligence. Radiographics : a review publication of the Radiological Society of North America, Inc. 2009;29(7):1897-906.

37. Morgan MB, Branstetter BFt, Lionetti DM, Richardson JS, Chang PJ. The radiology digital dashboard: effects on report turnaround time. J Digit Imaging. 2008;21(1):50-8.

38. Awan OA, van Wagenberg F, Daly M, Safdar N, Nagy P. Tracking delays in report availability caused by incorrect exam status with Web-based issue tracking: a quality initiative. J Digit Imaging. 2011;24(2):300-7.

39. International H. HL7 International 2019 [Available from: http://www.hl7.org/.

40. Library D. About DICOM 2019 [Available from: https://www.dicomlibrary.com/dicom/.

41. Kohli M, Dreyer KJ, Geis JR. Rethinking Radiology Informatics. American Journal of Roentgenology. 2015;204(4):716-20.

42. Teather D, Morton BA, du Boulay GH, Wills KM, Plummer D, Innocent PR. Computer assistance for C.T. scan interpretation and cerebral disease diagnosis. Stat Med. 1985;4(3):311-5.

43. Khorasani R. Clinical decision support in radiology: what is it, why do we need it, and what key features make it effective? J Am Coll Radiol. 2006;3(2):142-3.

44. Bates DW, Kuperman GJ, Wang S, Gandhi T, Kittler A, Volk L, et al. Ten commandments for effective clinical decision support: making the practice of evidence-based medicine a reality. Journal of the American Medical Informatics Association : JAMIA. 2003;10(6):523-30.

45. Ayaz A, Yanartaş M. An analysis on the unified theory of acceptance and use of technology theory (UTAUT): Acceptance of electronic document management system (EDMS). Computers in Human Behavior Reports. 2020;2:100032.

46. Batucan GB, Gonzales GG, Balbuena MG, Pasaol KRB, Seno DN, Gonzales RR. An Extended UTAUT Model to Explain Factors Affecting Online Learning System Amidst COVID-19 Pandemic: The Case of a Developing Economy. Frontiers in Artificial Intelligence. 2022;5.

47. Wiki E. Usability and user experience surveys 2019 [updated 8/16/2019. Available from: http://edutechwiki.unige.ch/en/Usability_and_user_experience_surveys#UTAUT.

48. Cheeseman SE. Communication and collaboration technologies. Neonatal Netw. 2012;31(2):115-9.

49. Pimmer C, Mhango S, Mzumara A, Mbvundula F. Mobile instant messaging for rural community health workers: a case from Malawi. Glob Health Action. 2017;10(1):1368236.

50. Bautista JR, Lin TTC. Nurses' use of mobile instant messaging applications: A uses and gratifications perspective. Int J Nurs Pract. 2017;23(5).

51. Iversen TB, Melby L, Toussaint P. Instant messaging at the hospital: supporting articulation work? Int J Med Inform. 2013;82(9):753-61.

52. Rosset C, Rosset A, Ratib O. General consumer communication tools for improved image management and communication in medicine. J Digit Imaging. 2005;18(4):270-9.

53. Fratt L. PACS Powers the Enterprise. Health Imaging Insights in Imaging & Informatics [Internet]. 2007 10/21/2019. Available from: https://www.healthimaging.com/topics/advanced-visualization/pacs-powers-enterprise.

54. Philips Adds Options to PACS. Imaging Technology News [Internet]. 2007 10/21/2019. Available from: https://www.itnonline.com/content/philips-adds-options-pacs.

55. Grabb A. Early experience with electronic messaging tightly integrated within PACS. J Am Coll Radiol. 2011;8(2):141-2.

56. America IN. INFINITT PACS 2019 [Available from: https://www.infinittna.com/solutions/radiology/infinitt-pacs/.

57. Health IW. Merge PACS Innovative Reading Workflows for Enterprise Radiology 2019 [Available from: https://www.merge.com/Solutions/Radiology/Merge-PACS.aspx.

58. Carestream Health I. RIS Module Streamlined Productivity. 2018.

59. Saince. Saince Merge Enterprise PACS 2019 [Available from: https://www.saince.com/international-solutions/saince-enterprise-pacs/.

60. Medical S. Sectra PACS and RIS - Examples of supported radiology workflows: Communication 2019 [Available from: https://medical.sectra.com/product/sectra-radiology-pacs-ris/.

61. Corporation FHA. Synapse EIS Features 2019 [Available from: https://www.fujifilmusa.com/products/medical/medical-informatics/radiology/RIS/index.html#features.

62. HealthCare A. XERO Viewer All images, One View 2019 [Available from: https://global.agfahealthcare.com/us/enterprise-imaging/universal-viewer/.

63. McFarlane D. Comparison of four primary methods for coordinating the interruption of people in human-computer interaction. Hum-Comput Interact. 2002;17(1):63-139.

64. Bates M. Health Care Chatbots Are Here to Help. IEEE Pulse. 2019;10(3):12-4.

65. Laranjo L, Dunn AG, Tong HL, Kocaballi AB, Chen J, Bashir R, et al. Conversational agents in healthcare: a systematic review. Journal of the American Medical Informatics Association : JAMIA. 2018;25(9):1248-58.

66. Beveridge M, Fox J. Automatic generation of spoken dialogue from medical plans and ontologies. Journal of biomedical informatics. 2006;39(5):482-99.

67. Mesko B, Hetenyi G, Gyorffy Z. Will artificial intelligence solve the human resource crisis in healthcare? BMC Health Serv Res. 2018;18(1):545.

68. Breastfeeding Si. A virtual assistant to help doctors in their daily work 2016 [Available from: https://www.safeinbreastfeeding.com/safedrugbot-chatbot-medical-assistant/.

69. Gupta A, Li H, Sharda R. Should I send this message? Understanding the impact of interruptions, social hierarchy and perceived task complexity on user performance and perceived workload. Decis Support Syst. 2013;55(1):135-45.

70. Czerwinski M, Cutrell E, Horvitz E. Instant Messaging: Effects of Relevance and Timing. 2000.

71. Rao A, Kim J, Kamineni M, Pang M, Lie W, Succi MD. Evaluating ChatGPT as an Adjunct for Radiologic Decision-Making. medRxiv. 2023.

72. Rao A, Pang M, Kim J, Kamineni M, Lie W, Prasad AK, et al. Assessing the Utility of ChatGPT Throughout the Entire Clinical Workflow: Development and Usability Study. J Med Internet Res. 2023;25:e48659.

73. Şendur HN, Şendur AB, Cerit MN. ChatGPT from radiologists' perspective. Br J Radiol. 2023;96(1148):20230203.

74. Thirunavukarasu AJ, Ting DSJ, Elangovan K, Gutierrez L, Tan TF, Ting DSW. Large language models in medicine. Nat Med. 2023;29(8):1930-40.

75. Qualtrics. QualtricsXM 2019 [Available from: https://www.qualtrics.com/.

76. Sudraben. In: X-ray.jpg L, editor. wikimedia: Wikimedia; 2018.

77. Imaging OA. Jane_Doe_CBCT_NEW_Report. In: Jane_Doe_CBCT_NEW_Report.jpg, editor. http://www.orbitimaging.com/imaging-services/radiologist-interpretation/.

78. screen-0. In: screen-0.jpg, editor.

79. Hsu W. Capturing Data Elements and the Role of Imaging Informatics11/2/2019. Available from: http://amos3.aapm.org/abstracts/pdf/99-27434-359478-111844-1383861762.pdf.

80. Zoom Video Communications I. Zoom 2021 [Available from: https://zoom.us/.

81. Otter.AI. Otter.AI 2021 [Available from: https://otter.ai.

82. Krippendorff K. Content Analysis: An Introduction to Its Methodology: Sage; 2004.

83. International H. HL7 Version 2 Product Suite 2019 [Available from: https://www.hl7.org/implement/standards/product_brief.cfm?product_id=185.

84. Mildenberger P, Eichelberg M, Martin E. Introduction to the DICOM standard. Eur Radiol. 2002;12(4):920-7.

85. Fatehi M, Safdari R, Ghazisaeidi M, Jebraeily M, Habibi-Koolaee M. Data Standards in Tele-radiology. Acta Inform Med. 2015;23(3):165-8.

86. contributors W. Application programming interface: Wikipedia, The Free Encyclopedia; 2019 [updated 9/23/2019. Available from: https://en.wikipedia.org/w/index.php?title=Application_programming_interface&oldid=915084030.

87. contributors W. Representational state transfer: Wikipedia, The Free Encyclopedia; 2019 [updated 9/23/2019. Available from: https://en.wikipedia.org/w/index.php?title=Representational_state_transfer&oldid=916246930.

88. International H. FHIR Overview 2019 [updated 12/27/2019. Available from: https://www.hl7.org/fhir/overview.html.

89. Hussain MA, Langer SG, Kohli M. Learning HL7 FHIR Using the HAPI FHIR Server and Its Use in Medical Imaging with the SIIM Dataset. J Digit Imaging. 2018;31(3):334-40.

90. Kamel PI, Nagy PG. Patient-Centered Radiology with FHIR: an Introduction to the Use of FHIR to Offer Radiology a Clinically Integrated Platform. J Digit Imaging. 2018;31(3):327-33.

91. Erickson BJ, Langer SG, Blezek DJ, Ryan WJ, French TL. DEWEY: the DICOM-enabled workflow engine system. J Digit Imaging. 2014;27(3):309-13.

92. America TRSoN. RSNA Informatics RadLex 2019 [Available from: http://radlex.org/.

93. America TRSoN. Common Data Elements (CDEs) for Radiology 2019 [Available from: https://radelement.org/.

94. Huser V, Rasmussen LV, Oberg R, Starren JB. Implementation of workflow engine technology to deliver basic clinical decision support functionality. BMC Med Res Methodol. 2011;11:43.

95. Waite S, Scott JM, Legasto A, Kolla S, Gale B, Krupinski EA. Systemic Error in Radiology. AJR Am J Roentgenol. 2017;209(3):629-39.

96. Chakraborty S, Reed M, Rybicki FJ, Fraser J, Glanc P, Levesque J, et al. Clinical Decision Support in Computerized Providers' Order Entry for Imaging Tests in Canada. Can Assoc Radiol J. 2017;68(4):357-8.

97. Kruger JF, Chen AH, Rybkin A, Leeds K, Guzman D, Vittinghoff E, Goldman LE. Displaying radiation exposure and cost information at order entry for outpatient diagnostic imaging: a strategy to inform clinician ordering. BMJ Qual Saf. 2016;25(12):977-85.

98. Claret PG, Bobbia X, Macri F, Stowell A, Motte A, Landais P, et al. Impact of a computerized provider radiography order entry system without clinical decision support on emergency department medical imaging requests. Comput Methods Programs Biomed. 2016;129:82-8.

99. Vecellio E, Georgiou A. Integrating the Radiology Information System with Computerised Provider Order Entry: The Impact on Repeat Medical Imaging Investigations. Stud Health Technol Inform. 2016;227:126-31.

100. Pevnick JM, Herzik AJ, Li X, Chen I, Chithriki M, Jim L, Silka P. Effect of computerized physician order entry on imaging study indication. J Am Coll Radiol. 2015;12(1):70-4.

101. Lee CI, Khodyakov D, Weidmer BA, Wenger NS, Timbie JW, Brantley B, et al. JOURNAL CLUB: Radiologists' Perceptions of Computerized Decision Support: A Focus Group Study From the Medicare Imaging Demonstration Project. AJR Am J Roentgenol. 2015;205(5):947-55.

102. Chan SS, Francavilla ML, Iyer RS, Rigsby CK, Kurth D, Karmazyn BK. Clinical decision support: the role of ACR Appropriateness Criteria. Pediatr Radiol. 2019;49(4):479-85.

103. American College of Radiology A. ACR Appropriateness Criteria 2019 [Available from: https://www.acr.org/Clinical-Resources/ACR-Appropriateness-Criteria.

104. Gillies RJ, Kinahan PE, Hricak H. Radiomics: Images Are More than Pictures, They Are Data. Radiology. 2015;278(2):563-77.

105. Ibrahim A, Vallieres M, Woodruff H, Primakov S, Beheshti M, Keek S, et al. Radiomics Analysis for Clinical Decision Support in Nuclear Medicine. Seminars in nuclear medicine. 2019;49(5):438-49.

106. Rizzo S, Botta F, Raimondi S, Origgi D, Fanciullo C, Morganti AG, Bellomi M. Radiomics: the facts and the challenges of image analysis. Eur Radiol Exp. 2018;2(1):36-.

107. Webopedia. ETL - Extract, Transform, Load 2019 [Available from: <https://www.webopedia.com/TERM/E/ETL.html>.
